# Dynamics and control of sister kinetochore behavior during the meiotic divisions in *Drosophila* spermatocytes

**DOI:** 10.1371/journal.pgen.1007372

**Published:** 2018-05-07

**Authors:** Soumya Chaurasia, Christian F. Lehner

**Affiliations:** Institute of Molecular Life Sciences (IMLS), University of Zurich, Zurich, Switzerland; College de France CNRS, FRANCE

## Abstract

Sister kinetochores are connected to the same spindle pole during meiosis I and to opposite poles during meiosis II. The molecular mechanisms controlling the distinct behavior of sister kinetochores during the two meiotic divisions are poorly understood. To study kinetochore behavior during meiosis, we have optimized time lapse imaging with *Drosophila* spermatocytes, enabling kinetochore tracking with high temporal and spatial resolution through both meiotic divisions. The correct bipolar orientation of chromosomes within the spindle proceeds rapidly during both divisions. Stable bi-orientation of the last chromosome is achieved within ten minutes after the onset of kinetochore-microtubule interactions. Our analyses of *mnm* and *tef* mutants, where univalents instead of bivalents are present during meiosis I, indicate that the high efficiency of normal bi-orientation depends on pronounced stabilization of kinetochore attachments to spindle microtubules by the mechanical tension generated by spindle forces upon bi-orientation. Except for occasional brief separation episodes, sister kinetochores are so closely associated that they cannot be resolved individually by light microscopy during meiosis I, interkinesis and at the start of meiosis II. Permanent evident separation of sister kinetochores during M II depends on spindle forces resulting from bi-orientation. In *mnm* and *tef* mutants, sister kinetochore separation can be observed already during meiosis I in bi-oriented univalents. Interestingly, however, this sister kinetochore separation is delayed until the metaphase to anaphase transition and depends on the Fzy/Cdc20 activator of the anaphase-promoting complex/cyclosome. We propose that univalent bi-orientation in *mnm* and *tef* mutants exposes a release of sister kinetochore conjunction that occurs also during normal meiosis I in preparation for bi-orientation of dyads during meiosis II.

## Author summary

For production of oocytes and sperm, cells have to complete meiosis which includes two successive divisions. These divisions convert diploid cells with a maternal and a paternal copy of each chromosome into haploid cells with only one copy of each chromosome. Chromosome copy reduction requires regulation of sister kinetochore behavior during the meiotic divisions. Kinetochores are protein networks assembled at the start of divisions within the centromeric region of chromosomes. They provide attachment sites for spindle microtubules which in turn exert poleward pulling forces. During pre-meiotic S phase, each chromosome is duplicated into two closely associated sister chromatids. At the start of the first meiotic division, both sister chromatids together assemble only one functional kinetochore, permitting subsequent separation of paired homologous chromosomes to opposite spindle poles. In contrast, at the onset of the second meiotic division, each sister chromatid organizes its own kinetochore followed by separation of sister chromatids to opposite spindle poles. To analyze when and how sister kinetochores are individualized, we have improved time lapse imaging with *Drosophila* spermatocytes. Our analyses in normal and genetically altered spermatocytes suggest that the release of sister kinetochore conjunction occurs during the first meiotic division after activation of the anaphase promoting complex/cyclosome.

## Introduction

The success of meiosis depends on regulation of sister kinetochore (KT) behavior during the two meiotic divisions. At the beginning of the first meiotic division, the two sister KTs function as a single unit. As a result, each of the two chromosomes in a bivalent, which results from pairing of homologous chromosomes before meiosis I (M I), is equipped with one KT. The two KTs of a bivalent permit its bipolar integration into the spindle and segregation of homologous chromosomes to opposite spindle poles during M I. In contrast, the two sister KTs behave as independent functional units during meiosis II (M II). Therefore, pairs of sister KTs are bi-oriented within the spindle during M II, permitting segregation of sister chromatids onto opposite poles.

The mechanisms enforcing a distinct behavior of sister KTs during M I and M II are still poorly understood. Progress has been made primarily with yeast where meiotic cells are more accessible than in animals and plants. In budding yeast, the monopolin complex is required for sister KT mono-orientation during M I [1–3]. It appears to function as a physical clamp that keeps the two sister KTs in close proximity [4–6]. The monopolin complex is restricted to *Saccharomycotina* species with point centromeres specified by a *CEN* DNA consensus sequence. In fission yeast, where regional centromeres are specified epigenetically, Rec8 and Moa1 were identified as being important for sister KT mono-orientation during M I [7, 8]. Rec8 is a meiosis-specific cohesin subunit [9]. During M I in fission yeast, Rec8 cohesin can also be detected within the centromere core regions. These centromeric Rec8 cohesin complexes appear to enforce tight cohesion of sister centromeres so that these jointly organize only one single KT [8, 10]. During M II, however, Rec8 cohesin is largely absent from centromere cores. The two sister centromeres are therefore presumably free to organize two functionally independent sister KTs in M II [8, 10]. While Moa1 appears to be required for maintenance of Rec8 within the core centromere during M I, it remains unclear how this M I-specific centromeric cohesin is established initially. Similarly, it is not known how Rec8 cohesin is removed from core centromeres before M II. Rec8 and Moa1/Meikin homologs have been identified in animal and plant species. They also appear to contribute to sister KT mono-orientation during M I [11–14], but their precise function in this process is also not understood.

Analysis of KT function during meiosis in complex multicellular organisms is more demanding compared to yeast. Plant and animal centromeres usually reside within extended highly repetitive genome regions that diverge rapidly during evolution. Presently it is still impossible to study animal meiotic divisions with large numbers of cells expanded and synchronized in vitro, because meiosis is embedded as a protracted process into the differentiation of highly specialized sex-specific gametes. In females, the asymmetry of the meiotic divisions preserves the large size of the oocyte and its maternal provisions. Only one of the four haploid meiotic products survives, while the three other nuclei are discarded with minimal cytoplasm as polar bodies. Moreover, animal oogenesis is usually accompanied by centrosome elimination. Chromosome segregation during female meiosis is therefore accomplished by acentrosomal spindles. In addition, progression through female meiosis is characterized by developmental arrests for co-ordination with fertilization. In contrast, male meiotic divisions are continuous and symmetric, using centrosomal spindles for the generation of four haploid nuclei that all differentiate into functional gametes. In case of dimorphic X and Y chromosomes, male meiosis has to achieve a regular segregation of these often highly unequal sex chromosomes. Many profound sex- and species-specific differences therefore impinge on KT behavior during meiotic divisions in animals.

Time lapse imaging for the characterization of meiotic KT behavior in animals at high temporal and spatial resolution has been completed primarily with mouse oocytes during M I [15–17]. For further study of meiotic KT behavior and its control, we have developed preparations that permit efficient time lapse imaging with *Drosophila melanogaster* spermatocytes. Thereby comparison between M I and M II is readily possible.

*D*. *melanogaster* spermatocytes were used early on for analyses of meiotic KT behavior. Pioneering serial section electron microscopy (EM) has provided a precise ultrastructural description [18, 19]. Accordingly, a hemispherical (HS) KT structure is present on each half-bivalent at the start of M I (S1 Fig). In this HS structure, the two sister KTs cannot be differentiated individually. However, at later M I stages, a double disc structure was observed, presumably representing the two sister KTs resolved in a side-by-side (SS) arrangement. The timing of the HS to SS transition was not determined precisely, as analysis of large cell numbers and their precise staging with serial section EM is difficult. Comparative EM analyses of KT morphology during M II have not been published. However, according to unpublished EM data mentioned in [20], the SS arrangement “is converted to a back-to-back arrangement as dyads enter the second meiotic division”. Despite minimal evidence it is often assumed that a back-to-back (BB) arrangement is established before the start of interactions between sister KTs and spindle microtubules (MTs) during M II [21, 22]. For geometric reasons, such an early transition from SS to BB, where sister KTs are on opposite faces of the dyad with chromatin in between, would favor an initial sister KT attachment to spindle MTs in the correct amphitelic manner. In contrast, the SS arrangement is predicted to increase the frequency of initial sister KT attachments of the erroneous syntelic type.

Correction of erroneous KT attachments is time-consuming and requires destabilization of attachments followed by re-attachment [23]. The spindle assembly checkpoint (SAC) [24], which is activated by unattached KTs, serves to delay anaphase onset, providing time for chromosome bi-orientation including error correction. In principle, a BB arrangement of KTs in combination with an appropriate extent of inherent KT attachment instability is sufficient to achieve an eventual orientation of all chromosomes in the correct bi-polar fashion within the spindle. However, mechanisms increasing KT attachment stability in response to mechanical tension were proposed to accelerate error correction. Tension generated by amphitelic KT attachment is predicted to be greater than that resulting from incorrect attachments. The stabilizing effect of tension on KT attachments has been demonstrated most convincingly by classic chromosome micromanipulation experiments with grasshopper spermatocytes [25].

Beyond grasshopper spermatocytes, those from *D*. *melanogaster* were also used early on for live analyses of progression through the meiotic divisions [26, 27]. Monitoring of chromosome movements by DIC or phase contrast microscopy followed by analysis of chromosome speed and directions has given crucial insight into the chromosome bi-orientation process [27–31]. However, KTs were not directly visualized in these studies.

Here, we describe meiotic KT behavior based on time lapse imaging with *D*. *melanogaster* spermatocytes and KT tracking during M I and M II. We demonstrate that chromosome bi-orientation is achieved with high comparable speed during both meiotic divisions, making the SAC dispensable. The duration from the start of interactions between KTs and MTs until bi-orientation of the last chromosome is about 100 fold shorter compared to M I in mouse oocytes [17]. Moreover, in contrast to this latter division, chromosome bi-orientation during both M I and M II does not involve multiple cycles of chromosome re-orientation within the spindle. Tension is shown to have a pronounced effect on KT attachment stability by analyses with mutants lacking homolog conjunction during M I. Remarkably, the high efficiency of chromosome bi-orientation in M II succeeds even though sister KTs are still in close spatial vicinity when their interactions with spindle MTs start. However, while not detectable by light microscopy during normal meiosis, we provide evidence from mutants suggesting that the sister KT conjunction which promotes mono-orientation during M I is released during the metaphase to anaphase transition of this first meiotic division.

## Results

### A timer reserves little but sufficient time for chromosome bi-orientation during meiotic divisions even in the absence of SAC function

**Fig 1:**
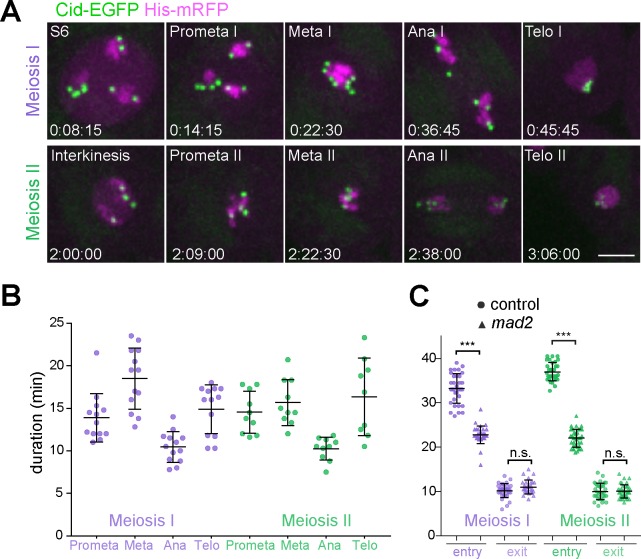
Temporal dynamics of the meiotic division program and its control by the SAC. **(A)** Progression through the meiotic divisions was analyzed by time lapse imaging with spermatocyte cysts expressing His2Av-mRFP and Cid/Cenp-A-EGFP. Representative still frames with a single spermatocyte illustrate the different division phases. Time (hours:minutes:seconds) is given with zero corresponding to start of the movie where spermatocytes were at the S6 stage. Only one daughter cell is shown in telophase. Scale bar = 5 μm. **(B)** Dot plots representing the duration of the distinct division phases during M I and M II in different cysts (n ≥ 9 cysts, each from a different testis) as well as the average durations (+/- s.d.). **(C)** Loss of SAC function accelerates anaphase onset. During M I and M II, the duration of entry (NEBD until anaphase onset) and exit (anaphase onset until interphase onset) was determined in control and *mad2* null mutants. Averages +/- s.d. are from n ≥ 29 cells from at least seven different testes.

To analyze progression through both meiotic divisions, we optimized time lapse imaging with *Drosophila* spermatocytes. As our approach preserves the integrity of spermatocyte cysts better than the most commonly applied protocols [27, 32], up to 16 cells progressing almost synchronously through M I and M II were imaged simultaneously (S1 Movie). For observation of centromeres and chromosomes, spermatocytes expressed Cid/Cenp-A-EGFP and histone His2Av-mRFP [33]. While earlier studies have reported the temporal dynamics of progression through M I [26, 27, 30, 31, 34, 35], information concerning interkinesis (IK) and M II has remained minimal. Progression through the complete process (M I, IK and M II) was observed to occur with similar dynamics not only in spermatocytes within a cyst (S1 movie), but also in different cysts. M I, IK and M II each lasted for close to one hour (average +/- s.d. = 58 +/- 6, 54 +/- 8, and 56 +/- 8 minutes, with n = 13, 11, and 9 cysts for M I, IK, and M II, respectively). The duration of the division phases (prometa-, meta-, ana- and telophase) were similar during the two meiotic divisions (Fig 1A and 1B).

In *Drosophila*, the SAC is not essential for viability and fertility, as indicated by the phenotype of *mad2* null mutant flies [36]. However, compared to controls, the fertility of *mad2* males is reduced to 50–64% of controls [36]. To assess the role of the SAC for the male meiotic divisions directly, we performed time lapse imaging with *mad2* null mutant spermatocytes. During both M I and M II, the interval from NEBD to anaphase onset was significantly shortened by about 10 minutes compared to controls, while the interval between anaphase onset and start of cytokinesis was not truncated (Fig 1C). Despite premature anaphase onset, chromosome segregation defects were very rare in the *mad2* mutants. Only one of 88 bivalent (in a total of 22 cells) failed to disjoin regularly during M I, and none of the 120 dyads (in a total of 30 cells) during M II. Therefore, although precocious, the start of anaphase I and II occurs after successful chromosome bi-orientation in *mad2* mutants.

### Assembly of spindles and kinetochores for physical interactions requires several minutes beyond nuclear envelope breakdown

Progression through the meiotic divisions in unperturbed spermatocytes is remarkably rapid, and further acceleration in *mad2* mutants is still compatible with regular chromosome segregation. Chromosome bi-orientation within M I and M II spindles must therefore succeed quickly. To study the bi-orientation efficiency in detail, we analyzed spindle and KT assembly, as well as the interactions between spindle MTs and KTs by time lapse imaging.

**Fig 2:**
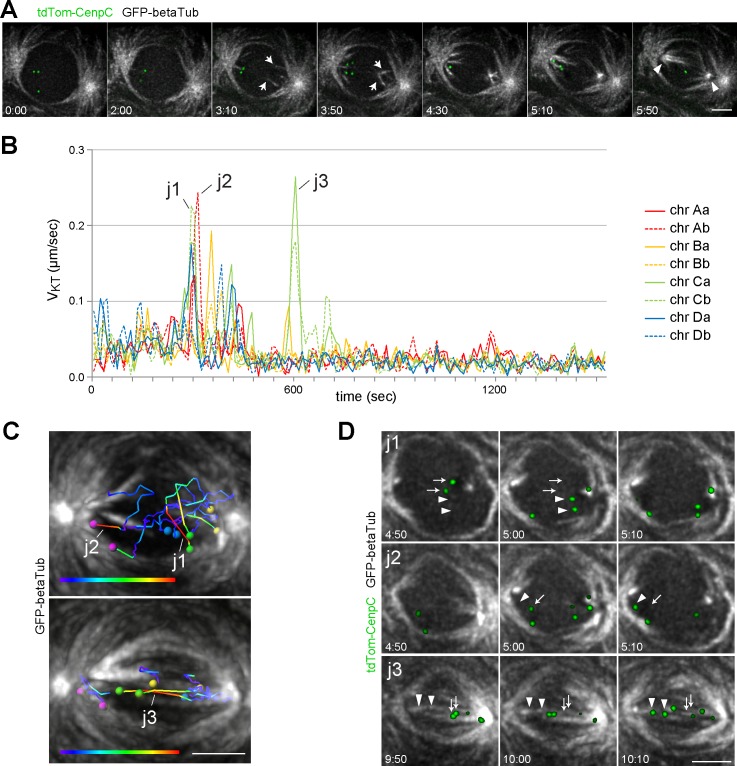
Spindle assembly and MT interaction with KTs. Time lapse imaging was performed with spermatocytes expressing GFP-βTub56D (GFP-betaTub) and tdTomato-Cenp-C (tdTom-CenpC) **(A)** Maximum intensity projections of optical sections (3–4 with 300 nm spacing) containing the pole to pole axis at the indicated times (minutes:seconds after NEBD I) are displayed. Arrows indicate initial formation of intranuclear MTs that are eventually bundled into the poles of the intranuclear spindle (arrowheads). **(B)** All KTs of a spermatocyte were tracked during M I (NEBD until end of metaphase) and their velocity (V_KT_) was plotted. The four bivalents were labeled with a capital letters (chr A-D) and the two KTs of a bivalent with small letters (a and b). The prominent KT jumps (j1-3) are further documented in panels C and D. **(C)** KT tracks representing the prominent jumps of KT pairs (j1-3) indicated in panel B. The early jumps (j1 and j2) are displayed in the top panel and a late jump (j3) in the bottom panel. In both panels, a still frame from the time point just after the jump is combined with KT tracks covering the preceding period of 15 time points (150 seconds). Tracks are displayed with a color code reflecting V_KT_ from slow (blue) to fast (red). The KTs are indicated by spheres with pairs displayed in the same color as also used in the V_KT_ curves (B). (**D)** GFP-βTub56D signals in the slices containing the jumping KTs (j1-3) are displayed at three consecutive time points (minutes:seconds after start of imaging) with maximum intensity projections (2–3 optical sections with 300 nm spacing). Arrows indicate KT positions prior to and arrowheads those after the KT jump. Scale bars = 5 μm.

Spindle formation in *Drosophila* spermatocytes has been studied in considerable detail (see for example [35, 37]), but only during M I. Spermatocytes expressing GFP-βTub56D [38] and His2Av-mRFP were used for a comparison of spindle behavior during MI and MII, which was found to be similar (S2 Fig). Of interest with regard to chromosome bi-orientation efficiency during meiosis, MTs and chromosomes started to interact with a delay of several minutes after nuclear envelope breakdown (NEBD). At NEBD I and II, as revealed by the sudden loss of the diffuse nucleoplasmic His2Av-mRFP signal, centrosomes were fully separated to opposite sides and associated with prominent cytoplasmic MT asters. However, MTs remained excluded from the nuclear interior for several minutes. NEBD is known to be incomplete during the meiotic divisions in spermatocytes [39]. Limited polar fenestration of the nuclear envelope occurs underneath the centrosomes, but in the lateral regions the nuclear envelope persists throughout M I and M II. Three to five minutes after NEBD, intranuclear MTs became detectable most prominently in foci close to the cytoplasmic centrosomes (S2 Fig; see also Fig 2A). As reported earlier [35], prominent MT bundles were also formed far away from these inner spindle poles and from chromosomes (S2 Movie), in stark contrast to female meiosis where acentrosomal MT nucleation is tightly chromosome associated [40, 41].

To study the dynamics of interactions between spindle MTs and KTs, we performed time lapse imaging with spermatocytes expressing GFP-βTub56D and tdTomato-Cenp-C [42]. Position and orientation of the first intranuclear MTs detected after NEBD I indicated that most of these MTs grew out neither from the cytoplasmic centrosomes nor from the KTs (Fig 2A). Rapid focusing of the growing intranuclear MTs created intranuclear spindle poles. Progressive bundling at both outer and inner poles caused MTs to pivot inwards and orient increasingly along the spindle axis (Fig 2A).

Tracking all eight KTs over time in 3D revealed that the original relative KT constellation was largely kept for about five minutes after NEBD I with only concerted and limited KT movements (Fig 2B). Subsequently, the initial KT constellation was broken up by rapid jumps of a single KT, partially followed by the partner KT on the same bivalent (Fig 2B–2D). These KT jumps presumably resulted from transport along MTs that have come into contact with one KT of the jumping pair. Unfortunately, such contacting MTs were often not clearly detectable in case of the initial KT jumps (Fig 2D, j1 and j2). Our detection sensitivity appears to be insufficient for reliable visualization of single or few bundled MTs. In contrast, prominent MT bundles adjacent to jumping KT pairs were detected during later stages (Fig 2D, j3), but the high MT density precluded an unequivocal identification of those MTs that served as tracks during the KT jumps. During final congression into the metaphase plate, KT pairs were always associated with obvious MT bundles, which grew stronger throughout metaphase and terminated in an end-on attachment at the KT (S2 Fig). Analyses of M II gave analogous results (S3 Fig). The first intranuclear MTs were again not associated with KTs. Initial KT jumps also occurred in the absence of evident contacting MTs and increasingly strong MT bundles with end-on attachment to the KT developed during metaphase.

To confirm that the observed KT jumps indeed reflect interactions between MTs and KTs, we sought to analyze KT tracks after spermatocyte-specific inactivation of KTs by transgenic RNAi. First, we characterized the expression of KT proteins known to be essential for mitotic KT function in spermatocytes. Imaging of green fluorescent variants of Spc105/Knl-1, Mis12, and Nuf2 [43, 44] demonstrated that these KMN network components [24] are expressed and recruited to centromeres also during the male meiotic divisions (S4 Fig). As with intranuclear MTs, assembly of the KMN network, which permits MT end-on attachments to KTs, required more than five minutes beyond NEBD to reach completion during both M I and M II (S4 Fig).

**Fig 3:**
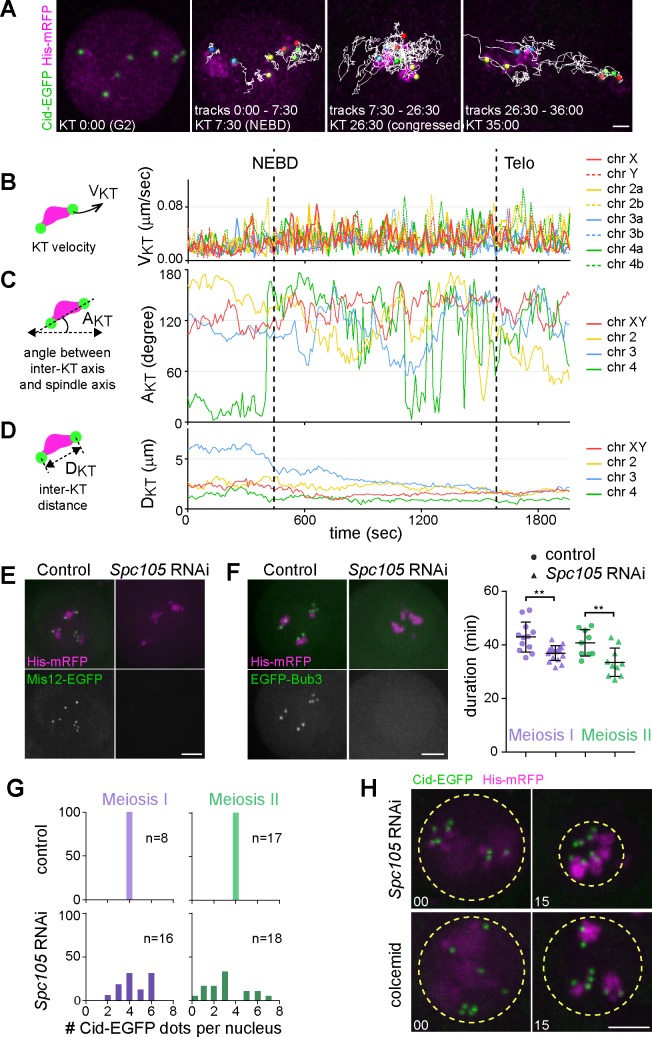
Progression through M I in the absence of KT function. **(A-D)** The kinetochore protein Spc105 was depleted in spermatocytes expressing Cid-EGFP and His2Av-mRFP. After time lapse imaging, all eight KTs in a representative spermatocyte were tracked during M I. **(A)** The KT tracks during consecutive division phases (time in minutes:seconds) are displayed as an overlay on still frames from the end of the corresponding phases. The two KTs associated with the same bivalent are represented by colored spheres with red, yellow, blue and green indicating chr XY, 2, 3 and 4, respectively. Transport of bivalents into the central region followed by aberrant segregation of unseparated KT pairs to opposite poles along horizontal axis is apparent. Scale bar = 3 μm. **(B)** KT velocities (V_KT_) plotted over time. **(C)** Angles (A_KT_) between the spindle axis and the axis connecting the two KTs of a bivalent plotted over time. **(D)** Distances (D_KT_) between the two KTs of a bivalent plotted over time. **(E)** Spc105 is required for recruitment of Mis12 to the KT. Spc105 was depleted in spermatocytes expressing Mis12-EGFP and His2Av-mRFP. Still frames after live imaging document the time point in prometaphase I where Mis12-EGFP signals at KTs are maximal in controls. **(F)** Spc105 is required for recruitment of Bub3 to the KT and for SAC function. Spc105 was depleted in spermatocytes expressing EGFP-Bub3 and His2Av-mRFP. Still frames after live imaging document the time point in prometaphase I where EGFP-Bub3 signals at KTs are maximal in controls. Dot plots indicate the duration of M I and M II as well as averages (+/- s.d., n ≥ 10 cells from at least six different cysts) in spermatocytes without (control) and with Spc105 depletion (*Spc105* RNAi). **(G)** Cid-EGFP dot counts per nucleus in daughter cells generated by M I and M II, respectively. Four dots per nucleus are observed in normal cells (control) and variable numbers after Spc105 depletion (*Spc105* RNAi). (n) number of analyzed cells. **(H)** Chromosome transport into the central region is evident after Spc105 depletion (*Spc105* RNAi) but not in the presence of colcemid. Representative still frames at NEBD I (00:00) and 15 minutes later are shown with dotted yellow circles indicating the region occupied by chromosomes. Scale bars = 5 μm.

Spermatocyte-specific depletion was found to be most efficient in case of Spc105. Spc105 depletion was performed with spermatocytes expressing Cid-EGFP which allowed KT tracking. The spermatocytes also expressed His2Av-mRFP. Therefore, moving KTs were accompanied by chromosomal His2Av-mRFP signals of characteristic shapes, which made KTs tracking more reliably compared to spermatocytes with fluorescent MTs instead of fluorescent chromosomes. Moreover, the His2Av-mRFP signals also permitted the reliable identification of those two KTs that were associated as a pair with the same chromosomal unit (bivalent in MI, dyad in MII). This in turn permitted extraction of additional parameters beyond KT speed (V_KT_), namely the angle (A_KT_) between the line defined by the KT pair and the spindle axis, as well as the distance (D_KT_) between the two KTs of a pair (Fig 3B–3D). Yet another advantage of His2Av-mRFP expressing spermatocytes was the possibility to identify each of the five distinct chromosomes (chr Y, X = 1, 2, 3, and 4) (S1 Text, S5 Fig, S3 and S4 Movies), creating an opportunity to detect chromosome-specific aspects of the bi-orientation process.

Spc105 depletion clearly abolished the rapid KT jumps that were observed in controls where they start about five minutes after NEBD I (compare Figs 2B and 3B). Therefore, the KT jumps depend on KT function.

### Spindle assembly contributes to chromosome congression in a kinetochore-independent manner

While Spc105 depletion effectively abolished the rapid KT jumps characteristic of normal prometaphase, time lapse imaging revealed considerable residual congression of chromosomes into the central region as well as subsequent poleward chromosome movements during exit from M phase during both M I (Fig 3A, S5 Movie) and M II (S6 Fig). Several observations indicated that these chromosome movements were not a result of residual KT function. Spc105 depletion completely eliminated the recruitment of the KT component Mis12-EGFP to the centromere (Fig 3E). Likewise, recruitment of the SAC component Bub3-EGFP was abolished and progression through MI and MII was accelerated (Fig 3F), comparable to *mad2* mutants. In addition, the poleward moving chromosomes were not always led by centromeres (S5 Movie) as during normal anaphase. Homologs failed to disjoin effectively during MI and moved together to the same pole (Fig 3A and S5 Movie). Centromere counts clearly confirmed chromosome missegregation after Spc105 depletion. While the normal number of eight Cid-EGFP dots per nucleus was still observed at the start of M I, this division generated daughter nuclei with a highly variable number of Cid-EGFP dots instead of the normal number of four (Fig 3G). During M II, sister chromatids also failed to disjoin effectively after Spc105 depletion (S6 Fig). Abnormal numbers of Cid-EGFP dots were also present after M II (Fig 3G).

Given that Spc105 depletion appears to inactivate KTs completely, the observed residual chromosome congression into the central region is likely caused by KT-independent interactions of spindle MTs with chromosomes. Such interactions might also contribute to the poleward movements of chromosomes during exit from M phase after Spc105 depletion, although cytoplasmic flow caused by contractile furrow activity during cytokinesis might be the main driver of these poleward movements. To assess the role of MTs, we performed KT tracking after addition of the microtubule inhibitor colcemid. This drug prevented meiotic spindle formation effectively, caused Bub3-EGFP persistence on KTs and substantial SAC-dependent delays during M I and M II (S7 Fig). In fact, colcemid addition to spermatocytes in culture revealed a greater SAC robustness than previously described based on feeding of colcemid to adult flies [34]. Importantly, in the presence of colcemid, chromosomes did no longer congress during M I (Fig 3H and S6 Movie) and M II (S6 Fig). Thus, normal spindle assembly drives a central concentration of chromosomes independent of KT function, as indicated by the chromosome behavior displayed after Spc105 depletion. The MT growth and bundling at the poles during the onset of the meiotic divisions presumably sweeps the bivalents from their initial widely spaced positions close to the nuclear envelope towards the central region rather than into an equatorial ring as in mammalian oocytes and somatic mitosis [45, 46]. Stable bi-orientation is established during a subsequent phase of KT-MT interactions. As expected, colcemid also prevented the rapid KT jumps characteristic of this second phase (S7 Fig).

### Bi-orientation of bivalents is completed with high efficiency

**Fig 4:**
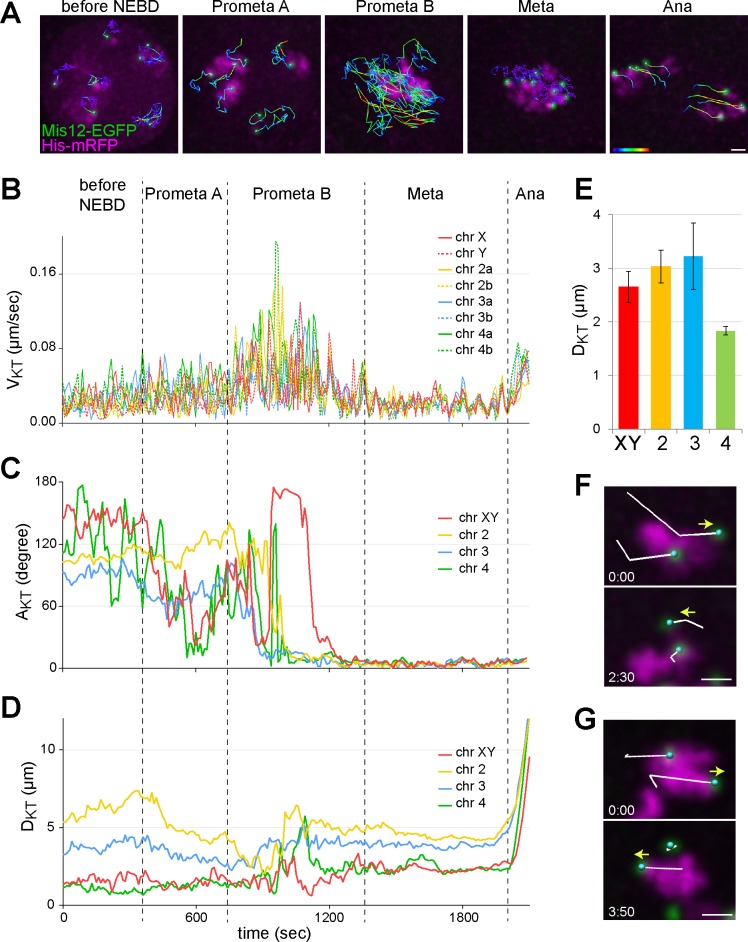
Efficiency of chromosome bi-orientation during unperturbed M I revealed by KT tracking. **(A-D)** All KTs of a representative spermatocyte expressing Mis12-EGFP and His2Av-mRFP were tracked during M I and assigned to chromosomes. **(A)** The KT tracks observed during the indicated phases are displayed as an overlay on the still frames from the end of the corresponding phases. Tracks are displayed with a color code reflecting V_KT_ from slow (blue) to fast (red). **(B)** V_KT_ plotted over time. **(C)** A_KT_ plotted over time. **(D)** D_KT_ plotted over time. Black dotted lines indicate division phase transitions. **(E)** Average D_KT_ at late metaphase I (+/- s.d., n = 6 cells). D_KT_ in case of the small chromosome 4 is less than in other bivalents (XY, chr 2 and chr 3). **(F, G)** Two distinct events of re-orientation of a KT pair during M I are illustrated with still frames oriented so that the spindle axis is horizontal. The KT tracks over the preceding two time points are superimposed on the images. Yellow arrows indicate the direction of movement of the re-orienting KT, which is to the right in the top panel and to the left after re-orientation in the bottom panel. The time interval separating the two still frames is indicated (minutes:seconds) Scale bars = 2 μm.

The effects of Spc105 depletion and colcemid addition demonstrated clearly that the rapid KT jumps, which started eventually after NEBD I during unperturbed M I, indicated interactions between MTs and KTs. To monitor how these interactions culminated in stable bi-orientation of bivalents, we examined V_KT_, A_KT_ and D_KT_ during unperturbed M I (Fig 4 and S3 Movie). Their temporal dynamics precisely revealed not only the onset of KT-MT interactions but also the final bi-orientation. The most prominent V_KT_ peaks were confined to a period starting with the first KT-MT interactions and ending with the stable bi-orientation of the last bivalent (Fig 4B). During metaphase, V_KT_ values were low, followed by a limited increase during anaphase (Fig 4B). The A_KT_ curves provided additional information on the dynamics of bi-orientation because during final bi-orientation of a bivalent its A_KT_ values approached zero and persisted at minimal levels (Fig 4C), a behavior abolished by Spc105 depletion (Fig 3C). In case of D_KT_, the values for different bivalents varied largely before NEBD I, although with a trend reflecting chromosome size, being minimal in case of the small chromosome 4 (Fig 4D, see also Fig 3D). Chromosome condensation around the time of NEBD I was accompanied by a decrease in D_KT_ values, in particular in case of the large chromosome bivalents (chr XY, 2 and 3). While after Spc105 depletion a slow D_KT_ decrease continued throughout M I (Fig 3D), stable bi-orientation during unperturbed M I was accompanied by an obvious although limited increase in D_KT_ (Fig 4D). During normal metaphase I, the average separation of stably bi-oriented KT pairs was maintained at a level roughly twofold larger than after Spc105 depletion. D_KT_ values during normal metaphase I were correlated with chromosome size (Fig 4E). KT separation in case of the small chromosome 4 bivalent was only about half of that observed in the other bivalents (Fig 4E). As expected, D_KT_ greatly increased during normal anaphase (Fig 4D), but not after Spc105 depletion (Fig 3D).

Based on the V_KT_-, A_KT_- and D_KT_ curves obtained from unperturbed spermatocytes, prometaphase I (i.e., the period between NEBD and bi-orientation of the last chromosome) is comprised of two sub-phases: (1) prometaphase A before the onset of KT-MT interactions, and (2) prometaphase B, the period starting with the initial MT-KT interactions and ending with the stable bi-orientation of the last bivalent (Fig 4B–4D). On average, prometaphase A lasted for 5 +/- 1.5 min (n = 6 spermatocytes from 2 distinct cysts) and prometaphase B for 9.75 +/- 3 min (n = 9 spermatocytes from 3 distinct cysts). As described further below, prometaphase II displayed a comparable subdivision.

The partially stochastic nature of the interactions between spindle MTs and KTs can lead to KT attachments other than the correct bipolar kind. The correction of erroneous attachments requires time-consuming turn-over of KT attachments. To assess the frequency of error correction, we examined the entire bi-orientation process of each bivalent within a total of five spermatocytes and determined the number of KT re-orientation events. A re-orientation event was only scored, when a particular KT was observed to jump suddenly towards one of the two spindle poles, followed at a later time by a jump towards the opposite pole, as illustrated by the examples in Fig 4F and 4G. In each of the five cells, one such KT re-orientation event was detected. Moreover, during only one of these events, the partner KT was observed to become pulled slightly away from the other KT towards the opposite pole in between the two antagonistically directed jumps of this other KT. Therefore, only this particular event appeared to represent a release after transient bi-orientation. The other events appeared to be cases of incomplete attachments where one of the two KTs of a bivalent lost a transient connection to one of the spindle poles, while the partner KT had no interactions with spindle poles. Although our analysis did not detect cases where transient interactions between KTs and spindle poles were not accompanied by evident changes in KT movements (direction, speed), our results indicated that the correct bipolar integration of bivalents into the M I spindle did not involve several cycles of bi-orientation and release. In Drosophila spermatocytes, the period from first MT-KT contacts until final bi-orientation including error correction is remarkably short and lasts less than 10 minutes.

### Stable attachment of univalents to spindles during M I is delayed compared to bivalents

In principle, the correction of erroneous KT attachments can succeed without sensing of mechanical tension at KTs [23]. However, if tension sensing is exploited, the need for repeated bi-orientation attempts is lower and the overall speed of bi-orientation is faster. The remarkable speed and efficiency of bi-orientation in *Drosophila* spermatocytes suggested that tension sensing was likely involved. Initial transient unipolar attachments of bivalents appear to drive KT jumps, orienting the inter-KT axis along the spindle axis, thereby favoring formation of correct bipolar connections. These in turn might lead to rapid attachment stabilization by generation of mechanical tension. The obvious increase in KT fiber strength during final congression of bivalents into the metaphase plate is consistent with these suggestions. To evaluate the role of tension, we decided to analyze *mnm* mutant spermatocytes. *mnm* function is required in spermatocytes for conjunction of homologs for M I [47]. The univalents present in *mnm* mutants are not expected to attain stable end-on attachment during M I, if this depends on the mechanical tension that normally results from bi-orientation of bivalents. Accordingly, in *mnm* mutants the rapid poleward jumps of KTs and associated chromosomes characteristic of prometaphase B are predicted to persist rather than stop within 10 minutes as in normal M I.

**Fig 5:**
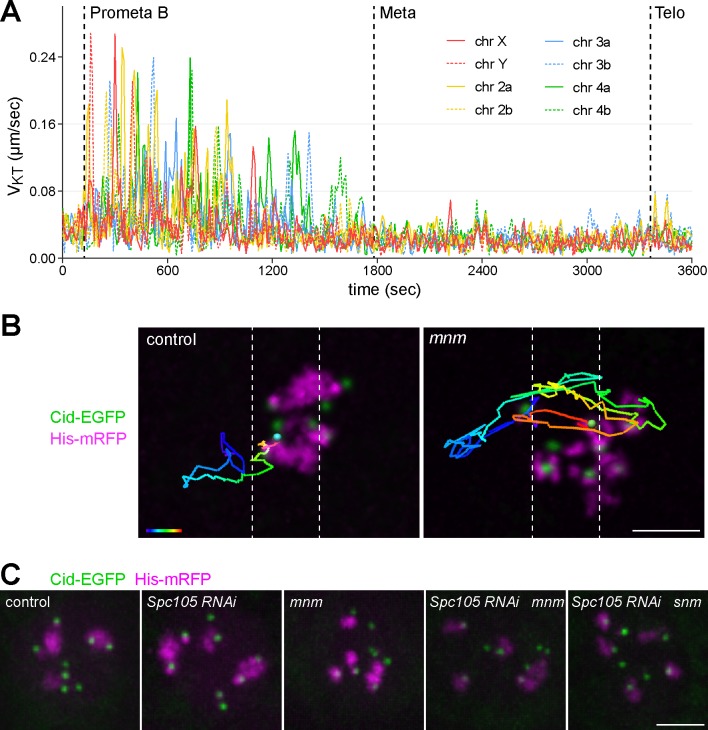
Behavior of KTs of univalents in *mnm* mutants during M I. After time lapse imaging with spermatocyte cysts from *mnm* mutant flies expressing Cid-EGFP and His2Av-mRFP, all eight KTs were tracked and assigned to chromosomes. **(A)** V_KT_ values plotted over time from a representative spermatocyte. Onset of the indicated phases marked by dotted lines. Metaphase onset was scored when the rapid KT jumps vanished rather than after bi-orientation of the last bivalent as in controls. **(B)** Comparison of KT tracks in control (left) and *mnm* mutants (right). The track of one representative KT from NEBD until metaphase onset is displayed as an overlay on the still frame from metaphase onset. Track color corresponds to time from early (blue) to late (red). White dotted lines demarcate the metaphase plate region (width 3 μm corresponding to about ¼ of the pole-to-pole distance). Scale bar = 3 μm. **(C)** State of sister KT conjunction during M I after Spc105 depletion in spermatocytes with and without *mnm* and *snm* function. The normal number of eight Cid-EGFP dots was detected by time lapse imaging with spermatocytes from the indicated genotypes. Still frames from representative spermatocytes during early prometaphase I are displayed. Scale bar = 5 μm.

Time lapse imaging with *mnm* mutant spermatocytes confirmed the expected homolog conjunction defect (Fig 5 and S7 Movie). Although some bivalents appeared to be still intact at NEBD I, their conversion into univalents occurred no later than at the start of prometaphase B. Subsequently, rapid KT-led jumps of univalents persisted in *mnm* mutant spermatocytes over a period that was definitely longer than normal prometaphase B (Fig 5A, compare with Fig 4B). In *mnm* mutant spermatocytes, a given KT was observed to make more excursions (i.e., a trip form the polar to the equatorial region, or vice versa) compared to controls (Fig 5B). On average, there were seven excursions per KT pair in *mnm* mutants and two in controls (n = 5 cells for each genotype). These results indicate that the rapid bi-orientation of bivalents during normal M I depends on effective stabilization of KT attachments to spindles by mechanical tension.

Interestingly, the rapid jumps of univalents in *mnm* mutants did not continue throughout M I. KT speed decreased throughout prometaphase B (Fig 5A) and after about 25 minutes, KT positions were just as stable as during normal metaphase I (Fig 4B). Moreover, a considerable number of KTs and associated univalents were within the equatorial region of the *mnm* mutant cells during this metaphase-like period. Eventually, a delayed exit from M I occurred in *mnm* mutants, as revealed by chromosome decondensation and cytokinesis. Univalent segregation into the daughter cells was largely random during exit from M I in *mnm* mutants, as expected [47].

### Mnm and Spc105 are not essential for sister kinetochore conjunction during M I

Sister KT conjunction during MI, which is of paramount importance for the correct separation of homologs, is poorly understood at the molecular level. However, Spc105 has been suggested recently to be important for conjunction of sister KTs [48]. In Spc105 depleted oocytes, a significantly higher number than the expected normal number of eight centromere signals was detected during M I [48]. In contrast, our time lapse imaging with Spc105 depleted spermatocytes did not reveal abnormal numbers of centromere signals. Spc105 depleted spermatocytes had the normal number of seven to maximally eight Cid-EGFP dots in mature spermatocytes before NEBD I (Fig 3A) [49] and eight dots thereafter (Fig 5C; n = 18 spermatocytes). In principle, the apparent differential Spc105 requirement for sister KT conjunction during female and male M I, respectively, might be explained by the alternative homolog conjunction system (AHC) which is active exclusively in males [47]. While our *mnm* mutant analysis confirmed that the AHC, like Spc105, is not essential for sister KT conjunction before M I (Fig 5C) [47], we considered the possibility that Spc105 and AHC might make independent contributions to sister KT linkage, resulting in functional redundancy. To evaluate this notion, Spc105 was depleted in *mnm* mutant spermatocytes expressing Cid-EGFP and His2Av-mRFP. But even in these spermatocytes the number of Cid-EGFP dots before and during M I was found to be normal (Fig 5C; n = 18 spermatocytes from at least three different testes for each genotype). Normal numbers of Cid-EGFP dots were also observed after Spc105 depletion in *snm* mutant spermatocytes, which have an apparently identical AHC defect as *mnm* mutants [47] (Fig 5C; n = 25 spermatocytes from at least three different testes). These results argue against a redundant involvement of Spc105 and AHC in sister KT conjunction during M I.

### Conjunction of sister kinetochores does not preclude their transient separation

**Fig 6:**
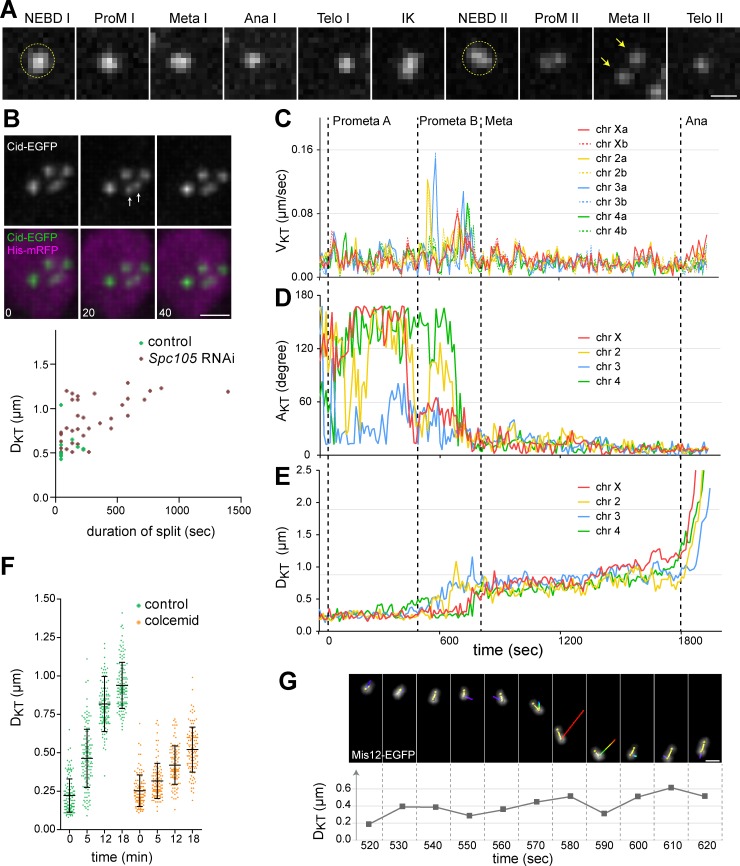
Sister KTs separation and bi-orientation during M II. **(A)** Sister KT behavior during progression through the meiotic divisions as observed by time lapse imaging with spermatocytes expressing Cid-EGFP and His2Av-mRFP is illustrated by representative still frames displaying the Cid-EGFP signal associated with one pair of sister KTs (except for telophase II where a single sister KT is shown). Sister KTs are detected as a single Cid-EGFP dots (yellow circle) from NEBD I to NEBD II until unequivocal separation in metaphase II (yellow arrows). **(B)** Time lapse imaging revealed occasional transient splitting of Cid-EGFP dots (white arrows), as illustrated with an event during IK. Time (seconds) is indicated. Scale bar = 2 μm. The scatter plot describes separation width and duration of such events in the indicated genotypes (n = 13 in controls, n = 36 after *Spc105* RNAi). **(C-E)** KTs in spermatocytes expressing His2Av-mRFP and Mis12-EGFP were tracked during M II followed by plotting of V_KT_ **(C)**, A_KT_ **(D)** and D_KT_ **(E)** over time. Black dotted lines mark the onset of the indicated division phases. **(F)** Spermatocytes expressing Mis12-EGFP and His2Av-mRFP were used for time lapse imaging of M II in the absence (control) and presence of colcemid. Inter sister KT distances (D_KT_) were measured at the indicated time points (minutes after NEBD II) and plotted with averages (+/- s.d.). **(G)** Separation of a sister KT pair during prometaphase II is documented with high magnification views (top) and a graph (bottom) of the distance between the two sister KTs (yellow line in stills) at the indicated time points (seconds after NEBD II). A track overlay in the top panel describes the displacement of one sister KT from the preceding to the displayed time point. Track color indicates V_KT_ from slow (blue) to fast (red). Spindle axis is vertical. After 620 sec, the sister KT pair remained stably bi-oriented during congression into the metaphase plate (located at the upper edge of the images) until anaphase. Scale bar = 0.7 μm.

While sister KT conjunction is crucial for M I, it needs to be resolved for M II. During M I, the transition in sister KT organization from HS to SS (S1 Fig) that has been suggested by the EM studies should not be detectable by light microscopy, as the gap between the two KT discs in SS configuration appears to be few nanometers only [18]. In contrast, a subsequent potential SS to BB transition might be detectable by our time lapse imaging. However, with Cid-EGFP expressing spermatocytes, we did not detect any obvious change in Cid-EGFP dot appearance during progression through M I (Fig 6A), not even with STED microscopy (S8 Fig). Likewise progression through IK did not include a persistent Cid-EGFP dot reorganization. Even at NEBD II, Cid-EGFP dots were sometimes elliptic but never permanently split into two signal maxima. Only later, an unequivocal separation of sister KTs was clearly apparent during metaphase II (Fig 6A and S8 Fig).

Intriguingly, time lapse imaging revealed occasional transient splitting of Cid-EGFP dots into two long before metaphase II (Fig 6B). Such “breathing” events were observed in mature spermatocytes before NEBD I, as well as throughout M I and IK. Frequency, duration and maximal separation distance of splitting events could be analyzed most readily during IK where centromere positions are relatively stable. Thus, 23 secondary spermatocytes (i.e., 92 Cid-EGFP dots) were tracked throughout IK (Fig 6B). A total of 13 splitting events were observed, i.e., about 0.17 per Cid-EGFP dot per hour. While clear separation into two distinct EGFP maxima was only apparent during a few frames usually (i.e., for about 20–40 seconds), a minority lasted longer (up to 3 minutes) (Fig 6B). The maximal spatial separation between the two fluorescence maxima was around 550 nm (Fig 6B). The actual frequency of sister centromere breathing is presumably somewhat higher because microscopic resolution was insufficient for detection of splitting along the z axis. However, assuming that the transient Cid-EGFP dot splitting represents sister centromere separation, we conclude that sister KTs are conjoined in a dynamic manner during M I and IK rather than by rigid persistent interlocking.

To assess a potential involvement of Spc105 in sister centromere breathing, we repeated our analyses after Spc105 depletion. This revealed a slight increase in frequency (0.3 per Cid-EGFP dot per hour, n = 150 Cid-EGFP dots tracked throughout IK), in duration and maximal separation of splitting events (Fig 6B). Since Spc105 is not detectable at the centromere during IK (S4 Fig), the effect of Spc105 depletion on sister centromere breathing during IK is presumably an indirect consequence of its absence during the preceding M I. By an analysis of splitting events during exit from M I (anaphase I and telophase I), we found that Spc105 depletion did not have a more pronounced effect during the stages where Spc105 is normally present. In controls, 28% of a total of 112 Cid-EGFP dots displayed a splitting event during exit from M I, compared to 58% of a total of 128 Cid-EGFP dots after Spc105 depletion. Moreover, Spc105 depletion in *mnm* mutant spermatocytes did not further increase the fraction of Cid-EGFP dots displaying transient splitting (50%, n = 224). Our observations suggest that Spc105 contributes to the dynamic coupling of sister KTs during the male meiotic divisions. Spc105 might assist in recruitment or protection of hypothetical dynamic sister centromere tethers. In Spc105 depleted spermatocytes, fewer tethers might be present, permitting more extensive sister centromere breathing.

### Closely spaced sister kinetochores are bi-oriented efficiently during M II

The extent of spatial separation between sister KTs when their interactions with spindle MTs start in M II is predicted to influence the frequency of erroneous initial attachments. Syntelic initial attachments necessitating correction will be numerous with closely spaced sister KTs displayed on the same chromosome face (i.e., in a SS organization). In contrast, widely separated sister KTs with shielding chromatin in between (i.e., in a BB organization) should preclude such attachments.

To determine the extent of sister KT separation at the start of the bi-orientation process in M II, we performed time lapse imaging with spermatocytes expressing His2Av-mRFP and Mis12-EGFP. Mis12 is more peripheral in mitotic KTs compared to Cid which is localized within the inner KT plate [43]. The pair of Mis12-EGFP dots generated from two sister KTs in a BB configuration is therefore more widely spaced apart and hence better resolvable than that obtained with Cid-EGFP [43]. KT tracking during M II resulted in V_KT_ curves that were very similar to those observed during M I (compare Figs 6C and 4B). Rapid KT jumps (i.e., prometaphase B) started again several minutes after NEBD II (Fig 6C) and were also dependent on KTs and MTs as indicated by analyses after Spc105 depletion and colcemid addition (S6 Fig). The A_KT_ curves observed during M II were also comparable to those from M I (Fig 6D, compare with Fig 4C). However, in case of the D_KT_ curves, the initial phase during M II was distinct from M I (Fig 6E, compare with Fig 4D). Until the onset of prometaphase B during M II, sister KTs were not separated sufficiently to allow their unequivocal resolution as two distinct Mis12-EGFP dots. Instead, a single somewhat elliptic Mis12-EGFP signal was observed. Assuming that these signals represent a superimposition of two circular sister KT signals, the extent of the spatial sister KT separation was estimated by measuring the distance between the two centers of the assumed circles (Fig 6E and 6F). The resulting distance provides an approximate upper bound of sister KT separation. Statistical comparisons at different time points should reveal even relatively small changes in average sister centromere separation during M II (Fig 6F). A first time point was chosen at NEBD II. A next time point was selected five minutes later, i.e., just before KT-MT interactions set in. Additional time points were analyzed 12 and 18 minutes after NEBD II, when about 50% and 100% of the dyads, respectively, have reached bi-orientation. Thereby the separation between sister KTs was found to increase slightly already within the first five minutes after NEBD II (Fig 6F). Separation was further increased at 12 and 18 minutes after NEBD II (Fig 6F). At the last time point, the separation was so extensive that two distinct Mis12-EGFP maxima were clearly resolved. The average value observed at 18 minutes (950 +/- 100 nm, s.d., n = 44 KT pairs) describes the separation of sister KT after dyad bi-orientation in M II.

To address the dependence of sister KT separation on MTs during M II, we made analogous analyses with colcemid treated spermatocytes (Fig 6F). In these cells, sister KT separation was largely suppressed. However, a minor but statistically significant increase in sister KT separation was still apparent already five minutes after NEBD II (Fig 6F). We conclude that a marginal increase in sister KT separation independent of MTs occurs early in prometaphase II. Presumably this separation is linked to chromosome condensation. Nevertheless, at the start of KT-MT interactions sister KTs are still very closely spaced. The eventual BB arrangement evident in metaphase II arises as a result rather than in preparation for bi-orientation.

To address whether sister KT separation at the start of mitosis is more pronounced than at the onset of M II, we performed time lapse imaging with syncytial *Drosophila* embryos for comparison. Sister KT separation at NEBD and during metaphase was estimated to be marginally larger in mitosis compared to M II (about 16.5% and 18%, respectively; S9 Fig). Therefore, the arrangement of sister centromeres at the start of M II does not appear to be dramatically different from that in mitosis.

Despite close spacing of sister KTs at the onset of prometaphase B during M II, bi-orientation succeeded rapidly, as indicated by the V_KT_ -, A_KT_—and D_KT_ curves (Fig 6C–6E). In the cell documented in Fig 6C–6E, it took only five minutes between the first KT jumps and bi-orientation of the last dyad. Average duration was 7 +/- 2 min (s.d., n = 5 secondary spermatocytes from two cysts). To assess the frequency of error correction, we monitored the entire bi-orientation process of 20 dyad chromosomes (i.e., 5 cells) in detail. These analyses also provided further support for the role of MTs in sister KT separation. Sister KT separation usually started with a poleward jump led by one of the two sister KTs (Fig 6G). Before this first jump, sister KTs were closely associated (except for rare occasional breathing events). The jump brought the inter-sister KT axis in alignment with the spindle axes. Rapidly thereafter sister KTs established bi-orientation, as suggested by increasing spatial separation of sister KTs and congression into the metaphase plate where stretched sister KTs remained stably bi-oriented until anaphase onset (Fig 6C–6E). We observed only one unequivocal case of release of this initial bi-orientation and two additional, less certain cases. We conclude that despite initial proximity of sister KTs during M II, stable bi-orientation of the majority of dyads succeeds with remarkable efficiency and speed without cycles of transient bi-orientation and release.

### Sister centromere individualization during M I depends on the APC/C activator Fzy/Cdc20

**Fig 7:**
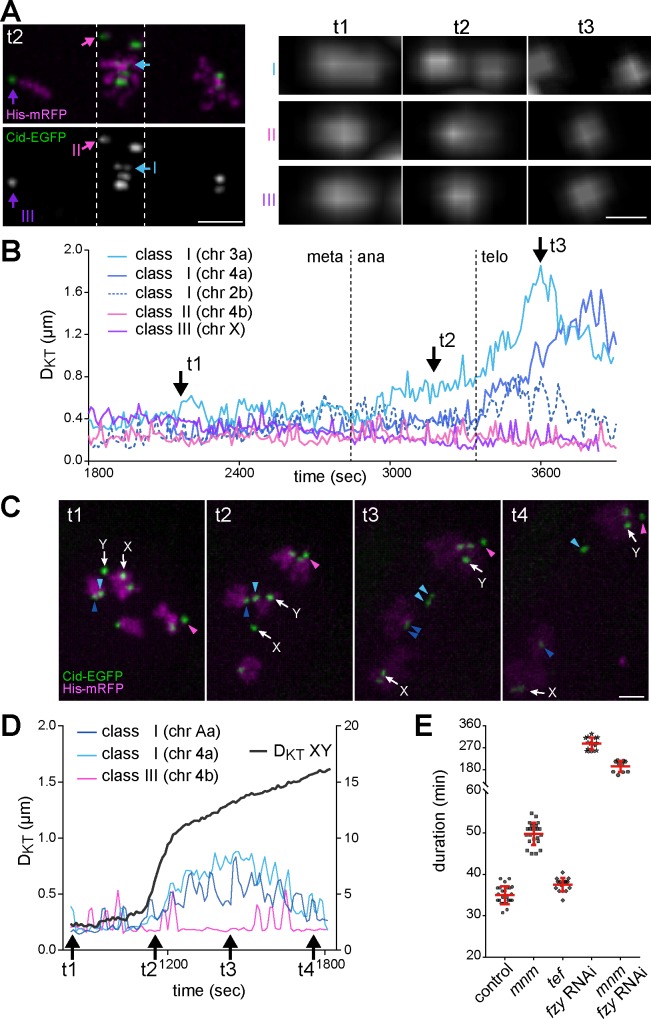
Bi-orientation of univalents and Fzy/Cdc20-dependent separation of their sister KTs during M I. For an analysis of the behavior of sister KTs associated with univalents during M I, time lapse imaging was performed with *mnm* **(A,B)** and *tef* **(C,D)** mutant spermatocytes expressing Cid-EGFP and His2Av-mRFP. **(A)** Univalents were assigned to three classes according to position and shape of the associated Cid-EGFP signals during exit from M I (left panel). Class I univalents have sister KTs within the metaphase plate and stretched apart, indicating bi-orientation. In class II univalents, sister KTs are also within the metaphase plate but unresolved, and class III univalents have unresolved sister KTs close to a spindle pole. High magnification views (right panel) display the Cid-EGFP signals of the indicated univalents (I, II, III) at different time points (t1, t2, t3; see also B). Scale bar = 3 μm (left panel) and 0.5 μm (right panel). **(B)** Distance between the two sister KTs of the indicated univalents plotted over time. The dotted lines mark the indicated division phase transitions. The arrows (t1, t2, t3) mark the time points displayed in panel A on the right. **(C)** Still frames from different time points (see also D). The KTs of the XY bivalent (white arrows) and of representative univalents (colored arrowheads) are marked. Scale bar = 2μm. **(D)** Distance between the two sister KTs of the univalents indicated in (C) plotted over time (y axis on the left), as well as separation of the KTs on the X and Y chromosome (D_KT_ XY) (y axis on the right). The rapid increase in D_KT_ XY reveals anaphase onset. The arrows (t1-t4) mark the time points displayed in (C). **(E)** The overall duration of M I was determined in spermatocytes with the indicated genotypes. Averages (+/- s.d.) are indicated in red. n > 10 cells from at least 3 different testes.

Although M I and IK are not accompanied by an evident spatial re-arrangement of sister KTs, the strikingly distinct behavior of sister KTs during M I and M II is inconceivable without at least some molecular changes. During M II, sister KTs become bi-oriented and stretched apart within minutes, while during M I they remain closely associated for mono-orientation. Interestingly, in contrast to unperturbed controls, *mnm* mutants provided microscopic evidence pointing to a transition in the state of sister KT conjunction occurring during progression through M I. This evidence was provided by some univalents that appeared to become bi-orientated during M I in *mnm* mutants. Such bi-orientation events became evident when KT movements slowed down eventually after the extended phase with rapid KT jumps in *mnm* mutants. After KT slow down, several univalents and their associated KTs remained stably within the equatorial region throughout a metaphase-like period (Fig 7A).

As Spc105 depletion had revealed KT-independent congression of bivalents, the stabilization of univalents within the equatorial region of *mnm* mutant spermatocytes might not necessarily indicate bi-orientation of sister KTs. However, a careful analysis of univalent behavior in *mnm* mutants argued for sister KT bi-orientation. A first class comprising about 30% of all the univalents (n = 224 univalents in 28 cells) remained stably within the equatorial region during the metaphase-like period, with an associated Cid-EGFP dot that was noticeably elongated along the spindle axis (Fig 7A), suggesting that sister KTs were being slightly stretched apart. The distance between sister KTs in these class I univalents during the metaphase-like period was on average around 330 nm, i.e., clearly below the distance observed during normal M II after dyad bi-orientation (720 nm; S9 Fig). Importantly, later during exit from M I in *mnm* mutant spermatocytes, the Cid-EGFP dot associated with class I univalents was split transiently but conspicuously into two clearly resolved dots along the spindle axis (Fig 7A and 7B). Some of the class I univalents displayed maximal separation of the two dots that was even larger than the normal sister KT separation during metaphase II (Fig 7B). While unequivocal scoring of a metaphase to anaphase (m/a) transition during M I was not possible in *mnm* mutant spermatocytes, chromosome decondensation revealed telophase I onset. Assuming that the m/a transition is about 10 minutes before telophase onset in *mnm* mutants, as in normal M I (Fig 1B), the Cid-EGFP dot splitting in class I univalents appeared to be concomitant with the m/a transition (Fig 7B). During this inferred m/a transition, Cid-EGFP dot splitting started in some of the class I univalents and after telophase onset it increased to a maximum (Fig 7B). At telophase onset, Cid-EGFP dot splitting became also detectable in additional class I univalents (Fig 7B). All univalents displaying clear Cid-EGFP dot splitting (class I) lagged severely during exit from M I. These findings suggested that class I univalents become bi-orientated within the M I spindle, presumably because sister KTs were linked to opposite poles. The pulling forces exerted by the spindle on these bi-oriented sister KTs apparently permitted the separation of sister KTs but only after m/a transition because this transition might be coupled with resolution of sister KT conjunction. Chromosome decondensation might remove additional tethers during telophase, allowing maximization of sister KT separation. Persisting pericentromeric sister chromatid cohesion presumably precludes complete sister KT separation and reinstalls close apposition of sister KTs after the collapse of pulling forces following disassembly of the spindle during telophase I. At the start of IK, sister KTs were again closely associated in *mnm* mutants. At NEBD II, the estimated sister KT separation was indistinguishable from controls (300 +/- 160 nm, s.d., n = 51 sister KT pairs from 4 distinct cells).

Apart from the class I univalents, there were others that also remained stably within the equatorial region during the metaphase-like period of *mnm* mutant M I. However, the associated Cid-EGFP signals in these class II univalents (38%, n = 96 univalents in 12 cells) were not split apart into two dots during exit from M I (Fig 7A and 7B). At telophase onset, class II univalents were usually transported slowly towards one of the poles during cytokinesis. Finally, class III univalents (32%, n = 96 univalents in 12 cells) also did not display Cid-EGFP dot splitting, but underwent a KT-led poleward movement early on, before the inferred m/a transition (Fig 7A and 7B). While class III univalents presumably started poleward movement after stable mono-orientation of the conjoined sister KTs, we assume that class II univalents remained stable within the metaphase plate because either one or both of the conjoined sister KTs had merotelic attachments to opposite spindle poles (rather than sister KTs linked to opposite poles as in class I). The number of class I, II and III univalents within a given *mnm* mutant spermatocyte was variable.

The temporal coincidence of the inferred m/a transition and the onset of Cid-EGFP dot splitting in class I univalents in *mnm* mutants suggested that sister KT individualization during normal M I might depend on APC/C activation. To monitor the m/a transition directly, we used *tef* mutant spermatocytes, in which conjunction of autosomal homologs but not of the XY chromosomes is defective [50]. The m/a transition during M I in *tef* mutants expressing His2Av-mRFP and Cid-EGFP was revealed by the rapid D_KT_ increase in the XY bivalent (Fig 7C and 7D, S8 Movie). Analysis of Cid-EGFP dot splitting in class I univalents in *tef* mutant spermatocytes (Fig 7C and 7D) clearly confirmed the notion that sister KT individualization is temporally coupled with the m/a transition in M I.

To evaluate whether the sister KT individualization revealed by the behavior of class I univalents in *mnm* mutants during M I depends on APC/C activity, we performed Fzy/Cdc20 depletion by spermatocyte-specific RNAi. Fzy is required for APC/C activity during M phase [51, 52]. Spermatocyte-specific Fzy depletion in otherwise normal spermatocytes resulted in the expected strong delay during metaphase I (Fig 7E, S9 Movie). After Fzy depletion, spermatocytes remained in metaphase for more than four hours (compared to 18 minutes in controls) followed by an abnormal M I exit. Importantly, after Fzy depletion in *mnm* mutant spermatocytes, Cid-EGFP dot splitting in class I univalents was strongly delayed. While Cid-EGFP dot splitting in class I univalents in *mnm* mutants without Fzy depletion occurred always within 50 minutes after NEBD I (n = 17 class I univalents in nine cells), it was never observed to happen within this time frame after Fzy depletion in *mnm* mutant cells (n = 21 cells). These results support the proposal that sister KT individualization depends on APC/C activity.

## Discussion

With *Drosophila* spermatocytes we have established efficient time lapse imaging of progression through both meiotic divisions at high temporal and spatial resolution. While broadly applicable, our focus here was primarily on KT behavior. We show that sister KTs are closely associated not only at the start of M I but also during exit from M I, IK and entry into M II. At NEBD II, sister KTs are so closely apposed that they cannot be resolved individually by light microscopy (including STED). Their later evident separation depends on interactions with MTs. In contrast to previous suggestions [20], therefore, an obvious BB geometry that would favor amphitelic over syntelic KT attachments to the spindle [21, 23] is not established in preparation but as a result of dyad bi-orientation in M II. Remarkably, despite close vicinity of sister KTs at M II onset, the bi-orientation process succeeds rapidly without repeated cycles of transient bi-orientation and release. In case of M I, where the bi-orientation process proceeds with comparable speed, its high efficiency is shown to depend on pronounced stabilization of KT attachments by mechanical tensions, as revealed by our analyses with *mnm* mutants. Interestingly, while sister KTs are predominantly in close association up to metaphase II, some underwent occasional transient dissociation episodes before and during M I, as well as during IK, indicating that sister KTs are not linked by a rigid persistent mechanical clamp, not even at M I onset. The apparent dynamic nature of sister KT conjunction presumably explains why univalents, as present during M I in *mnm* and *tef* mutants, eventually achieve bi-orientation occasionally. Interestingly, an abrupt splitting of the Cid-EGFP dot associated with such bi-oriented univalents is observed depending on APC/C activity at the m/a transition in M I. Therefore, we propose that the pulling forces acting on such bi-oriented univalents expose what is likely a regulated release of sister KT conjunction during progression through M I. This release of sister KT conjunction at the m/a transition might remain hidden during normal M I, as attachment of bivalents to spindles does not result in pulling forces that act in opposing directions on sister KTs.

Time lapse imaging of progression through the meiotic divisions at high temporal and spatial resolution in animals is challenging. Accurate KT tracking has been achieved only with mouse oocytes in M I [15–17]. Mouse testes have not yet been used for analyses of progression through M I and M II, although short term imaging of the rapid, telomere-led movements of chromosomes during meiotic prophase has been described [53]. With our optimized live imaging protocol, *Drosophila* spermatocytes should gain additional attraction. Intact cysts with 16 interconnected spermatocytes can be used for comprehensive KT tracking during near synchronous progression through both meiotic divisions. Comparison of the meiotic divisions in *Drosophila* spermatocytes with M I in mouse oocytes exemplifies evolutionary plasticity. Compared to mouse oocytes, M I in *Drosophila* males is tenfold faster and a symmetric division of a tenfold smaller cell with centrosomal spindles and far fewer bivalents that lack crossovers and re-orient rarely.

Sister KT mono-orientation during M I as a consequence of centromeric sister chromatid cohesion mediated by Rec8 cohesin and protected by Moa1/Meikin might be widely conserved [54]. In *Drosophila*, however, a canonical Rec8 cohesin complex does not exist [55, 56]. Instead, a functionally analogous complex containing the proteins Solo and Sunn is clearly required for sister KT mono-orientation during M I, as well as Ord, a potential loading factor of this complex [57–62]. However, the involvement of these proteins in sister KT linkage before MI might be indirect, as these proteins localize primarily to the pericentromeric region and maintain sister chromatid cohesion until late metaphase II.

Another *Drosophila* protein recently implicated in sister KT conjunction during M I is the KMN component Spc105/Knl-1 [48]. Spc105 depletion during oogenesis resulted in M I figures where sister KTs were mostly resolved individually instead of being fused into a single unit [48]. In contrast, as shown here, Spc105 depletion in spermatocytes did not have the same dramatic effect on sister KT linkage during M I. Incomplete depletion in our experiments is an unlikely explanation for the discrepancy, as KT function was observed to be completely abolished by our time lapse imaging. Spc105 depletion also eliminated SAC function during male meiosis. As M I in *Drosophila* oocytes is interrupted by an arrest in metaphase I, which lasts until fertilization and egg deposition, loss of SAC function after Spc105 depletion might actually compromise this metaphase I arrest and permit progression until sister KT individualization. While findings concerning Mps1 function in oocytes are consistent with a SAC involvement in temporal control of progression through M I [63], the precise role of this checkpoint for the metaphase I arrest in *Drosophila* oocytes remains to be studied carefully. Alternatively, the sex-specific effects of Spc105 depletion might indicate that sister KT linkage during female and male M I is mechanistically distinct.

Based on the behavior of sister KTs of bi-oriented univalents in *mnm* and *tef* mutants during M I (class I univalents), we propose that progression through the m/a transition in M I results in a release of sister KT conjunction in a Fzy/Cdc20-APC/C dependent manner. If correct, it would be important to identify the proteins targeted by Fzy/Cdc20-APC/C for the release sister KT conjunction during M I. Fzy/Cdc20-APC/C is known to target many proteins and it might act rather indirectly. The separase inhibitor securin is well-known target of Fzy/Cdc20-APC/C. After securin degradation, separase might cleave the alpha-kleisin of hypothetical centromeric cohesin complexes and thereby eliminate sister chromatid cohesion within the centromeric region. However, our previous analyses have suggested that separase activity is unlikely to be required for the release of sister KT conjunction during M I [64].

Because our readout for the state of sister KT conjunction during progression through M I is the sister KT separation distance in bi-oriented univalents, it is conceivable that Fzy/Cdc20-APC/C acts by increasing the pulling forces of the spindle rather than by changing the quality of sister KT conjunction. Although spindle forces are known to change during the m/a transition at least during mammalian mitosis [65], we consider this potential explanation to be less likely. In M II, the pulling forces exerted on bi-oriented normal dyads are strong enough to stretch sister KTs very noticeably apart already before the m/a transition. We assume that the pulling forces acting on bi-oriented class I univalents before the m/a transition during M I should produce the same noticeable sister KT separation, if the strength of sister KT conjunction in these univalents was identical to that in normal dyads during metaphase II. However, before the m/a transition in M I, sister KT separation in class I univalents was observed to be far less than in metaphase II dyads, while after the m/a transition it increased abruptly to values greater than those in metaphase II dyads. Evidently our arguments depend on the untested assumptions that forces exerted by M I and M II spindles are comparable and that also KT attachments just before the m/a transition are equivalent mechanically in case of bi-oriented class I univalents during M I and normal dyads during M II.

Our findings concerning the control of sister KT behavior during meiosis confirm and extend the results of elegant and sophisticated chromosome micromanipulation experiments with grasshopper spermatocytes [66, 67]. According to these studies, progression into anaphase I confers onto sister KTs the ability to bi-orient efficiently [66]. Moreover, despite acquisition of this bi-orientation ability, sister KTs remain in an SS configuration until conversion into a BB arrangement by spindle pulling forces during bi-orientation in prometaphase II [67].

One of the observations with grasshopper spermatocytes [67] does not appear to be in accordance with our findings in *Drosophila* spermatocytes. After detachment of bi-oriented grasshopper dyads from M II spindles, sister KTs remain in a BB configuration [67] arguing for an irreversibility of the SS to BB conversion. Plastic rather than elastic arrangement of sister KTs has also been observed in mammalian chromosomes during prometaphase in mitosis [68]. In contrast, our observations of sister KTs on bi-oriented univalents during exit from M I revealed an impressive elasticity of sister KT linkage. After spindle collapse, widely separated sister KTs were completely reunited into a state indistinguishable from that of normal sister KT pairs before bi-orientation in M II. It seems likely therefore that progression through IK or re-entry into M phase (i.e., M II) induces additional changes in the organization of sister KTs. In fact, we demonstrate that the distance between sister KTs increases over time during entry into M II even in the absence of MTs. This limited separation might reflect regulation of the activities of cohesin, condensin and topoisomerase II, as extensively documented in case of chromosome condensation during entry into mitosis [69].

The presence of sister KTs in a configuration that is more SS than rigid BB configuration at the start of M II is predicted to cause a heavy demand for error correction. Error correction involving sensing of the mechanical tension generated by correct bi-orientation is expected to be considerably more efficient than without tension sensing [23]. Our comparison of chromosome movements during M I in control and *mnm* mutants provides strong evidence for tension sensing in *Drosophila* spermatocytes. While normal bivalents, which are exposed to mechanical tension as a result of bi-orientation, develop stable end on attachments of their KTs to spindle MTs very rapidly, the univalents present in *mnm* mutants display a significantly prolonged period of transient attachment/detachment cycles, similar as univalent sex chromosomes resulting from a deletion eliminating most of the pairing site from the X chromosome in *Drosophila* [20] or in grasshopper X0 males [70]. We assume that effective stabilization of KT attachments by mechanical tension operates during M II as well, promoting an error correction efficiency that can cope with the unfavorable SS configuration of sister KTs at the start of this division.

While prometaphase B with its characteristic KT attachment/detachment cycles is clearly prolonged in *mnm* mutants during M I, eventually a metaphase-like period follows, where even mono-oriented univalents acquire stable KT attachments. Therefore, the stability of KT attachments increases over time in a tension-independent manner. An analogous temporal increase in KT attachment stability occurs during mitosis in cultured mammalian cells and *Drosophila* neuroblasts, as well as in mouse oocytes during M I [15, 71–75]. In somatic cells, the progressive stabilization of KT attachments during prometaphase reflects a decrease in Cdk1 activity resulting from early degradation of cyclin A which escapes inhibition by the SAC [71, 73, 76]. However, the opposite (a gradual increase in Cdk1 activity) achieves the same during the extraordinarily extended prometaphase I in mammalian oocytes [15, 75]. The stabilization of KT attachments during the much faster M I in *Drosophila* spermatocytes likely reflects a decrease in Cdk1 activity, since prometaphase B in *mnm* mutants is clearly prolonged by Fzy depletion.

As in mitosis, metaphase I is probably terminated by full activation of Fzy/Cdc20-APC/C causing securin degradation and consequential separase activation, as well as Cyclin B degradation, resulting in a precipitous drop and converse increase in Cdk1 and phosphatase activities, respectively. The precise dynamics of the initial partial decrease in Cdk1 activity and the relative timing of the m/a switch appear to be crucial determinants of the frequency of erroneous KT attachments. Accordingly, the differences in speed and frequency of univalent bi-orientation caused by mutations in *tef* and *mnm* without and with Fzy depletion might reflect differences in the dynamics of Cdk1 activity. In *mnm* mutants, the impossibility of normal tension-mediated stabilization of KT attachments clearly delays the m/a transition presumably because of delayed SAC silencing. Bi-orientation of univalents occurs at the end of this prolonged prometaphase B. Similarly, the chromosome micromanipulation experiments in grasshopper spermatocytes during M I have shown that normal M I chromosomes can be induced to attach in the M II manner eventually, but only with difficulty after repeated detachment from the spindle [66]. In *tef* mutants, where one bivalent forms normally (i.e., the XY bivalent) and the premature dissociation of others is relatively slow, the m/a transition was observed to be far less delayed compared to *mnm* mutants where univalents arise early in M I without the XY exception. The shorter delay of the m/a transition in *tef* mutants is paralleled by a far lower frequency of bi-oriented univalents. Fzy knockdown greatly delayed M I exit, but it did not result in a permanent arrest in M I, presumably because it was not entirely complete. After Fzy depletion in *mnm* mutants, prometaphase B was most strongly delayed. But also in this case, bi-orientation of univalents was observed eventually before exit from M I.

Evidently, the mechanisms responsible for sister KT conjunction before M I and its timely release before M II remain far from being clear. Clarification of this crucial meiotic process will likely profit from the experimental accessibility of *Drosophila* spermatocytes that includes efficient time lapse imaging as demonstrated here.

## Materials and methods

### *Drosophila* genetics

All the *Drosophila* lines with various mutations and transgenes that were used have been described previously (S1 Table). Lines with new combinations of these mutations and transgenes were generated by standard crossing and meiotic recombination. The genotypes of the analyzed testes are specified in detail in S2 Table.

### Testis preparation and time lapse imaging

Most previously published time lapse studies with *Drosophila* spermatocytes were performed after dissection of testis in halocarbon oil, followed by testis disruption for release of spermatocytes. In our hands, this approach caused frequent disintegration of spermatocyte cysts, which appeared to be linked with limited long-term viability, preventing completion of both meiotic divisions in vitro. In contrast, preparations in tissue culture medium were found to be readily compatible with successful completion of both meiotic divisions. The tissue culture medium was Schneider’s medium (ThermoFisher Scientific, #21720) supplemented with 10% fetal bovine serum (FBS) (Invitrogen) and 1% penicillin/streptomycin (Invitrogen, #15140). For most analyses, testes were dissected from early pupae that were still white or light brown. Exceptionally, testes were dissected from young adult males. Microscopically visible dominant marker mutations (*Cy*, *Tb*, *Dfd-EYFP*) were exploited for the identification of animals with the correct genotypes. With some markers, the correct genotype was selected during the larval stages followed by aging to the desired pupal stage.

Before testis dissection, tissue culture medium was pipetted into the cavities of a glass slide with three depressions. Pupae were opened in the first depression with a pair of forceps. The two testes with residual attached fat body were isolated using tungsten needles and washed in the second depression. After removal of fat body tissue, testes were transferred into the third depression. The procedure was repeated until 10–12 testes were ready. These were then transferred into a 45 μL drop of tissue culture medium in the center of a 35 mm culture dishes with glass bottom (MatTek Corporation, #P35G-1.5-14-C) and fitted with a peripheral ring cut from filter paper (Whatman, No. 1) wetted with 300 μL of water to reduce evaporation of the sample medium during imaging. Testes were disrupted gently with tungsten needles to disperse intact cysts. To dampen cyst mobility during subsequent imaging, 15 μL of 1% w/v methylcellulose (Sigma, #M0387) in tissue culture medium was pipetted on top of the drop with the dispersed cysts. After settling down, the methylcellulose also gently flattened the cysts. The lid of the dish was closed and sealed with a strip of parafilm before mounting the preparation on the microscope stage. Imaging was performed at 25°C in a room with temperature control using a spinning disc confocal microscope (VisiScope with a Yokogawa CSU-X1 unit combined with an Olympus IX83 inverted stand and a Photometrics evolve EM 512 EMCCD camera, equipped for red/green dual channel fluorescence observation; Visitron systems, Puchheim, Germany).

To monitor all spermatocytes within a cyst, a 40×/1.3 oil immersion objective was used for acquisition of image stacks with 30–40 sections and 800 nm spacing at 45 seconds intervals. For the comparison of the temporal dynamics of progression through the meiotic divisions in different genotypes and treatments, a 60×/1.42 oil immersion objective was usually used for acquisition of image stacks with 25–35 sections and 500 nm spacing at 45 seconds intervals. Typically the resulting stacks contained a large majority but not necessarily all of the spermatocytes of a cyst. For tracking chromosomes, KTs and MTs at maximal spatial and temporal resolution, a 100×/1.4 oil immersion objective was used for acquisition of image stacks with 40–45 sections and 300 nm spacing at 10 seconds intervals.

After imaging for 5–6 hours with stack acquisition at 45 seconds intervals, cysts could still be observed to enter into the meiotic divisions in our preparations. However, all the results described here were obtained with cysts that progressed through meiosis within 0–2 hours after the onset of imaging.

Drugs were administered by pipetting small volumes of stock solution (1 μL) directly to the final testis preparation in the 35 mm dishes. Colcemid (demecolcine, Sigma, #D6165) was dissolved in DMSO (10 mM) before further dilution in tissue culture medium and used at a final concentration of 10 μM.

### Stimulated emission depletion (STED) microscopy

Before fixation and immunofluorescent labeling, late larval and pupal testes (about 25) were dissected in testis buffer (183 mM KCl, 47 mM NaCl, 10 mM Tris-HCl, pH 6.8). Fat body tissue was removed gently without disrupting the testes. Testes were then transferred to a 15 μL drop of testis buffer on a poly-L-lysine-treated slide and cut open with a tungsten needle. Cysts were dispersed and flattened by placing a 22 mm x 22 mm cover slip on the drop. After two minutes of incubation, the preparation was frozen in liquid nitrogen. After flipping off the cover slip with a razor blade, the slide was immersed into ethanol for 10 minutes at -20°C. The region with the squashed testes was fixed by adding 0.4 mL of 4% paraformaldehyde in phosphate buffered saline (PBS) for seven minutes. Permeabilization was done twice in PBST-DOC (PBS containing 0.3% Triton-X100 and 0.3% sodium deoxycholate) for 15 min each. After a rinse in PBST (PBS containing 0.1% Triton X-100), blocking solution (PBST containing 5% fetal bovine serum) was added for 30 minutes. The primary antibody (rabbit anti-EGFP; Schittenhelm et al., 2007) was cleared by centrifugation for 20 minutes at 10,000 x g at 4°C before dilution (1:3000) in blocking solution. Incubation with primary antibody was done in a humid chamber overnight at 4°C, or for two hours at room temperature. After a quick rinse with PBST, slides were incubated in PBST for 15 minutes four times. Blocking before incubation with the secondary antibody was done for 30 minutes. The stock solution with the secondary antibody (goat anti-rabbit IgG conjugated to STAR 635P; Abberior) was also cleared by centrifugation, and after dilution in blocking solution (1:200), the incubation with secondary antibody as well as subsequent washing was performed as described above for the primary antibody. For DNA staining, Hoechst 33258 (1 μg/μL in PBS) was applied for five minutes followed by three washes with PBS. Finally, the preparation was mounted with a drop of Prolong diamond (ThermoFisher Scientific, P36965) and a cover slip. Before imaging, samples were left flat for curing at room temperature for at least 24 hours before imaging.

Image acquisition was completed with a Leica SP8 inverse STED 3X microscope equipped with a 100× objective. Spermatocytes during the meiotic stages were identified based on the DNA staining. Single sections were acquired with a three channel sequence. First, the DNA staining was imaged using a laser scanning confocal mode. Second, the anti-EGFP staining was acquired also with a confocal mode. Third, the anti-EGFP staining was imaged using a time gated STED mode with a pulsed 775 nm laser line for depletion.

### Image processing and analysis

Before image analysis, deconvolution was performed with some of the image stack sequences (Figs 2–7, S2 Fig, S3 Fig, S5 Fig, S6 Fig, S9 Fig) using Huygens Professional Deconvolution software (SVI, Netherlands) with a theoretical point spread function calculated by the software in combination with the classic maximal likelihood estimation algorithm.

For the analysis of the temporal dynamics of progression through the meiotic divisions, the time points of different meiotic phase transitions were scored by visual inspection of the time lapse sequences. Scoring was based on the analysis of the His2Av-mRFP signals. In addition, the relatively weak autofluorescent background signals generated by mitochondria in the green channel, which were clearly detectable after appropriate contrast enhancement, were exploited for scoring transition time points. Moreover, the specific EGFP signals generated by tagged centromere and KT proteins were considered as well. During M I and M II, NEBD, i.e., the prophase to prometaphase transition, was scored when the diffuse nucleoplasmic His2Av-mRFP signal started to drop precipitously. The prometaphase to metaphase transition was scored when the last pair of KT had congressed into the metaphase plate and had lost mobility. Metaphase onset could be scored particularly well after KT tracking and analysis of the three parameters V_KT_, A_KT_ and D_KT_ (see results). The metaphase to anaphase transition could be scored readily based on the KT movements. While the distance between a pair of KTs remains essentially stable throughout metaphase, it increases abruptly at anaphase onset. In M I, XY separation and occasionally also that of some other bivalents lagged somewhat behind. Anaphase onset was scored when the majority of bivalents started to separate. Scoring the anaphase to telophase transition could only be achieved with limited precision. When the KTs had reached the spindle poles (end of anaphase A), spindle pole separation (anaphase B) still continued with a very gradual subsequent slowdown. In comparison, the onset of contractile furrow activity, as revealed by the mitochondrial autofluorescence, occurred more abruptly and was therefore scored as telophase onset. Finally, the transition from telophase to interphase, was scored when chromatin masses had reached a perfectly round shape and the contractile furrow full constriction, as revealed by a gap between regions of mitochondrial autofluorescence in the two daughter cells. To assess the variability of the dynamics of progression through the meiotic divisions between different cysts (Fig 1B), three non-adjacent cells within a given cyst were randomly selected followed by determination of the duration of the division phases (prometa-, meta-, ana- and telophase) in these three cells. The three values for a given phase were averaged to arrive at a data point representing the cyst. A total of 13 and 10 cysts were analyzed during M I and M II, respectively.

Some mutations, RNAi—and drug treatments had the effect that certain transitions between the phases of the meiotic division could no longer be scored by applying the criteria described above. For the comparison of control with *mad2* mutants (Fig 1C) and *Spc105* RNAi (Fig 3F), the duration from NEBD to the m/a transition (“entry”) and from the m/a transition to onset of contractile furrow activity (“exit”) was determined. For some comparisons (Fig 7E and S7 Fig) an overall M I duration (from NEBD I until onset of chromosome condensation) was used.

For KT tracking we used the spot detection function of the software Imaris (Bitplane; version 7.7.2 and 8.3.0). During M I, the software readily identified the majority of KTs correctly when programmed to detect a sphere with a diameter of 500 nm. Inadvertently identified background artefacts were deleted and ignored KT signals were identified and added manually. Autoregressive tracking of the KT signals over time in image stack sequences acquired at the maximal spatial and temporal resolution (see above) resulted in tracks that were largely correct except during prometaphase B when the rapid KT jumps occur. However, with manual correction it was possible to generate tracks providing an accurate description of the movements of each KT except in rare cases of spermatocytes in which it was impossible to resolve the tracks of a few KTs over periods that lasted usually for less than four time points. Manual correction of tracks was successful because the associated His2Av-mRFP signals were also taken into account, while these were ignored by the software. Based on the associated His2Av-mRFP signals, the KT tracks were also assigned to specific chromosomes (see S1 Text and S5 Fig). While the identification of the chromosome 4 and XY bivalents during M I was unproblematic, discrimination and identification of the two large autosomal bivalents depended on clear His2Av-mRFP signals during early prometaphase. Moreover, during M II chromosome 2 and 3 could not be discriminated, while the other chromosomes X, Y and 4 were identifiable.

During M II, the two sister KTs were so closely associated that they could not be resolved individually until they were separated apart by bi-orientation. Therefore, the monitoring of individual sister KTs until metaphase II was impossible by spot detection and automatic tracking with the Imaris software, although they could be tracked readily as a pair. To arrive at an estimate for the distance between sister KTs over time during prometaphase II, an assumed position was therefore assigned manually for each sister KT using Imaris software. To assign these positions, the objects representing signals from sister KT pairs in 3D view along the microscopic z axis were inspected. Along the longest axis of these objects within the x y plane, two spheres were placed so that they fill the object optimally and their center points were used as approximate sister KT positions.

After exporting the KT positions obtained by tracking or manual assignment with Imaris software, the values for A_KT_ and D_KT_ (see results) were calculated using Excel (Microsoft Office). The V_KT_ values were provided by the Imaris software. The vector representing the spindle axis, which was used for the calculation of the A_KT_ values, was determined as follows. To each KT pair associated with either a bivalent during M I or a dyad during M II a KT vector connecting these two KTs was assigned. The origins of these KT vectors were chosen so that all these origins migrated to the same spindle pole during anaphase. The direction of the spindle vector was then calculated by averaging the direction of all KT vectors over a period of about 35 frames during metaphase. Occasionally, the spindle vector was also assigned manually to the spindle poles revealed by the KT movements during anaphase.

To determine the distance between sister KTs at selected time points during M II (Fig 6F), we used the function “Measurement points” of the Imaris software. For these analyses, a given signal representing a pair of sister KTs was visually inspected while rotating a three dimensional image reconstruction in space with the Imaris software. Thereby the longest axis of the signal object was identified, followed by filling the object optimally by manual positioning of two equal sized spheres along this axis. The sphere centers were used as an estimate of the sister KT positions and the distance between these positions was calculated by the Imaris software.

For the quantification of the KT signal intensities over time (S4 Fig and S7 Fig), we used the function “Isosurfaces” of the Imaris software for isolation of the KT signals by automatic segmentation. Parameters were selected so that the large majority of the centromeric signals were isolated. The pixel intensities were integrated over the volume comprised within all of the KT isosurfaces in a cell at each time point and exported into Excel for plotting.

In the figures, maximum intensity projections are displayed unless stated otherwise. These projections were generated in Imaris or Image J. Movies (avi files) were generated with Imaris. Graphs were generated with Excel or GraphPad prism. P values were calculated using a two tailed student t test (* = p < 0.05; ** = p < 0.01; *** = p < 0.001). Adobe Photoshop and Illustrator were used for figure assembly.

## Supporting information

S1 Table *Drosophila melanogaster* lines. (PDF)

S2 Table Sample genotypes. (PDF)

S3 Table Numerical data used for graphs and summary statistics. (XLSX)

S1 Text Chromosome identification during time lapse analysis of M I. (PDF)

S1 Fig Sister KT organization during progression through meiosis. Based on serial sectioning and electron microscopy with *Drosophila* spermatocytes [18], a single hemispherical structure (HS) is present at the start of M I, composed of the two sister KTs, which cannot be distinguished. Later in M I, a side-by-side arrangement (SS) is observed with two closely associated KT discs (i.e., the two sister KTs in all likelihood). It is assumed that sister KTs eventually adopt a back-to-back organization (BB) with inner centromere chromatin in between. The precise timing of the HS to SS and SS to BB transitions and their molecular basis are unknown.
(TIF)

S2 Fig Spindle dynamics and congression of bivalents. **(A)** The dynamics of spindle organization during the meiotic divisions was analyzed by time lapse imaging with spermatocytes expressing GFP-βTub56D (GFP-betaTub) and His2Av-mRFP (His-mRFP). Still frames showing a representative spermatocyte at the indicated stages. The intranuclear spindle poles which were most prominent during metaphase are indicated by arrowheads.
**(B)** Congression of bivalents into the metaphase plate was analyzed by time lapse imaging with spermatocyte cysts expressing GFP-βTub56D and tdTomato-Cenp-C. High magnification views document the onset of the final congression of a bivalent into the metaphase I plate. Maximum intensity projections of only those optical sections containing the two KTs of the chosen bivalent are shown at the indicated time points (minutes:seconds after NEBD I). Dotted white lines mark the initial KT positions for reference. One centrosome and the associated intranuclear spindle pole are visible on the left side. Spindle axis is horizontal. Congression onset is accompanied by the apparent disappearance of the connections (red arrows) between the left spindle pole and the merotelically attached KT on the right, while its connections to the right spindle pole remain (white arrows).
**(C)** Stably bi-oriented bivalents in metaphase I have KTs with end-on attachments to prominent MT bundles. A maximum intensity projection (MIP) of eight optical sections with 300 nm spacing is shown in the top panel, while MIPs of only two sections (section numbers as indicated) are displayed below. Scale bar = 3 μm.
(TIF)

S3 Fig Spindle assembly and interaction of MTs with KTs during M II. Time lapse imaging was performed with spermatocytes expressing tdTomato-Cenp-C and GFP-βTub56D. **(A)** Still frames at selected time points (minutes:seconds after NEBD II) illustrate progression until metaphase II in a representative spermatocyte. Each panel is a MIP of the z slices containing the spindle poles (3–4 optical sections with 300 nm spacing). Scale bar = 2 μm. **(B)** A single optical section documenting an example of a transient sister KT separation event during early prometaphase with an inter sister KT axis that is not oriented towards one of the spindle poles and without associated detectable MTs. **(C)** Later movements of KT pairs during prometaphase II can occur in association with MTs that are not necessarily oriented towards a spindle pole. Four consecutive z-sections are shown. Scale bar = 3 μm. **(D)** The four sister KT pairs were tracked in a representative cell from NEBD until metaphase, and the V_KT_ values were plotted over time. The first KT jump event (j1) is documented in panel E. **(E)** The top panel includes an overlay with KT tracks over the preceding four time points (40 seconds) which include the KT jump indicated in panel D (j1). Track colors reflect V_KT_ from slow (blue) to fast (red). The middle and bottom panels document localization of MTs and sister KT pairs before and after the jump, respectively. Arrows and arrowheads indicate the position of the KT that is leading during the jump, before and after the jump, respectively. Scale bar = 2 μm. **(F)** Stably bi-oriented dyads in metaphase have KT with end-on attachments to prominent MT bundles. Left most panel is a MIP comprising of eight optical sections with 300 nm spacing. MIPs of only a few sections (as indicated) are shown in the additional panels. Scale bar = 3 μm.
(TIF)

S4 Fig Dynamics of KT assembly during the meiotic divisions. Time lapse imaging with spermatocytes was performed for the analysis of the meiotic localization of KMN network proteins (Nuf2, Spc105 and Mis12) fused to EGFP. Spermatocytes also expressed His2Av-mRFP for monitoring progression through the meiotic divisions. Representative spermatocytes are shown at the indicated stages: spermatocytes at stage S6 (S6), NEBD, prometaphase, metaphase, anaphase, telophase, interkinesis (IK), and postmeiotic interphase (Post). **(A, D)** Spc105-EGFP. **(B, E)** Mis12-EGFP. **(C, F)** Nuf2-EGFP. Scale bar = 5 μm. **(G)** EGFP signal intensities at KTs from representative cells during entry into M I. Maximal intensity was set to 100 arbitrary units (a.u.). NEBD was used for curve alignment.
(TIF)

S5 Fig Identification of the five different chromosomes and KT assignment confirms higher levels of Cid-EGFP in the Y chromosome. **(A)** Appearance of chromosomes and associated Cid-EGFP signals during M I in XY and X0 spermatocytes. After Cid-EGFP and His2Av-mRFP time lapse imaging, four distinct bivalents could be differentiated and identified in spermatocytes with a normal XY karyotype during prometaphase I (for further explanations see S1 Text). The two Cid-EGFP dots of the chromosome 4 bivalent are indicated by orange arrowheads, those of the large autosomes (chromosome 2 and 3) by blue arrowheads. The prominent His2Av-mRFP blobs next to the KTs of chromosome 3 are indicated by red arrows. The Cid-EGFP dot of the X chromosome is indicated with a grey arrowhead, that of the Y chromosome (absent in X0) with a white arrow. During the metaphase to anaphase transition in XY spermatocytes (Meta/Ana I) the Cid-EGFP dot on the Y chromosome has minimal associated His2Av-mRFP signals. Time (seconds) is indicated in the upper right corner with zero corresponding to the first anaphase frame. Cid-EGFP intensities quantified after spot segmentation are color-coded from low (blue) to high (red) in the middle and bottom rows. Amounts of associated His2Av-mRFP are visualized by the white isosurfaces in the bottom row. Scale bars = 2 μm.
**(B)** Average intensity (+/- s.d.) of Cid-EGFP dots in XY (n = 4) and X0 spermatocytes (n = 3). While X, Y and 4^th^ chromosomes were identified, the two large autosomes (A1 and A2) were not distinguished as either chromosome 2 or 3. In each cell, the average intensity of the two Cid-EGFP dots associated the chromosome 4 bivalent was set to 100%. The difference between the X and the other KTs was highly significant (p < 0.0017, t test).
**(C)** Characteristic features of the XY bivalent. The Y chromosome contains only very low levels of His2Av-mRFP, and in the X chromosome His2Av-mRFP is primarily present in the centromere-distal euchromatic arm region. XY pairing is mediated by repeats within the rDNA loci of these chromosomes (NORs). Scale bar = 1 μm.
**(D)** Time lapse imaging was performed with spermatocytes that had a *lacO* repeat array on one of the two chromosome 2 homologs. Moreover, the spermatocytes expressed GFP-lacI-nls and His2Av-mRFP. A still frame from early prometaphase is shown. The prominent KT-proximal His2Av-mRFP blobs on the chromosome 3 bivalent are indicated by red arrows. Scale bar = 5 μm.
**(E)** Still frames from prometaphase II after time lapse imaging with spermatocytes expressing Cid-EGFP and His2Av-mRFP. The two large autosomes, which could not be discriminated, are indicated with blue and chromosome 4 with orange arrowheads. 50% of the prometaphase II cells contained the X chromosome (left panel, grey arrowhead) and the other 50% the Y chromosome (right panel, white arrow). Scale bar = 1 μm.
(TIF)

S6 Fig Effects of colcemid and Spc105 depletion on KT and chromosome movements during M II. **(A)** Time lapse imaging was performed with spermatocytes expressing Cid-EGFP and His2Av-mRFP after addition of colcemid. All eight sister KTs in a representative spermatocyte were tracked during the first 20 minutes of M II. V_KT_ values were plotted over time.
**(B,C)** Time lapse imaging was performed with spermatocytes expressing Cid-EGFP and His2Av-mRFP after spermatocyte-specific Spc105 depletion. **(B)** One sister KT in each dyad was tracked during progression through M II and V_KT_ values over time were plotted. Arrows (t1-t5) indicate the time points documented in (C). **(C)** Still frames at selected time points (see B) illustrate delayed sister KT separation and chromosome movements accompanying chromosome decondensation during exit from M II. Sister KTs co-segregated during these chromosome movements rather than move apart as during normal M II.
**(D)** Still frames from the indicated times (minutes after NEBD) after treatment with colcemid or Spc105 depletion indicate that spindle assembly can drive congression of dyads into the central region in a KT-independent manner. Yellow circles indicate the regions occupied by chromosomes. Scale bars = 3 μm (C) and 2 μm (D).
(TIF)

S7 Fig Effects of the microtubule inhibitor colcemid on progression through the meiotic divisions. **(A)** Time lapse imaging with spermatocytes expressing GFP-βTub56D and His2Av-mRFP demonstrated that addition of colcemid prevents spindle formation effectively. In contrast, spindle assembly proceeds normally in mock-treated spermatocytes. Time (minutes) is indicated in the lower left corner with zero corresponding to NEBD I. Scale bar = 10 μm.
**(B)** Colcemid delays the release of Bub3 from KTs and exit from M I. Spermatocytes expressing EGFP-Bub3 and His2Av-mRFP were used for time lapse imaging of progression through M I. In the absence of colcemid, EGFP-Bub3 signal intensities reach a peak at KTs in prometaphase and drop at a maximal rate in anaphase. In the presence of colcemid, EGFP-Bub3 disappearance from KTs and exit from M I are strongly delayed. Representative time points (minutes:seconds after NEBD) are illustrated by still frames. Analogous observations were made in all other spermatocytes analyzed (at least eight from two different cysts for each condition). Scale bar = 3 μm.
**(C)** Colcemid induces a strong delay during M I and M II. Colcemid was added just before either NEBD I or NEBD II. The duration of the meiotic divisions (NEBD until cytokinesis onset) was determined in control and *mad2* null mutant cells. Average durations +/- s.d. are from n ≥ 6 cells from at least six different testes.
**(D)** Colcemid eliminates rapid KT jumps during prometaphase and the stretching apart of KTs that accompanies chromosome bi-orientation during unperturbed meiosis. Time lapse imaging was performed with spermatocytes expressing Cid-EGFP and His2Av-mRFP after addition of colcemid. All eight KTs in a representative spermatocyte were tracked during M I. V_KT_ and D_KT_ values were plotted over time.
(TIF)

S8 Fig Analysis of sister KT associations during meiosis by STED microscopy. **(A-C)** Testis squash preparations of *Cid-EGFP* expressing spermatocytes were fixed and labeled with anti-GFP. Double labeling with a DNA stain (not displayed) allowed identification and staging of cells during the meiotic divisions. **(A)** Representative single optical sections through a given Cid-EGFP dot acquired by STED and confocal laser scanning (LSC) microscopy, respectively. All panels displayed at the same magnification except for prometa II. Scale bars = 1 μm. **(B)** Intensity curves along the yellow dotted lines in (A) confirm increased resolution of STED images. **(C)** Quantification of the fraction of Cid-EGFP dots with a circular (see for example (A) meta I), elongated (see for example (A) interkinesis) and resolved sisters (see for example (A) prometa II) appearance at the indicated stages.
(TIF)

S9 Fig Comparison of the distance between sister KTs at the start of M I, M II and mitosis. Identical settings were used for time lapse imaging with spermatocytes and embryos expressing Cid-EGFP and His2Av-mRFP. The distance between sister KTs was measured at NEBD and in metaphase. **(A)** Representative still frames are displayed. The two homologous centromere pairs are widely separated in bivalents during M I, as indicated by the yellow dotted line. In contrast, sister KTs cannot be resolved initially at the start of M II and mitosis, as indicated by the yellow dotted circles. Scale bar = 2 μm. **(B)** Dot plot representing individual measurements as well as average (+/- s.d.); at NEBD, n = 16 KT pairs (M I), 51 sister KT pairs (M II) and 42 sister KT pairs (mitosis); at metaphase, n = 48 KT pairs (M I), 42 sister KT pairs (M II) and 51 sister KT pairs (mitosis). Differences between average separation were statistically significant (t test): (*) p = 0.024, (***) p = 0.0058.
(TIF)

S1 Movie Progression of a *Drosophila* spermatocyte cyst through the meiotic divisions. Spermatocyte cysts were released from dissected testes expressing Cid/Cenp-A-EGFP and His2Av-mRFP. Spermatocytes within a cyst are linked via cytoplasmic bridges through ring canals and progress through the meiotic divisions almost synchronously. MIPs of image stacks with 18 focal planes spaced by 1 μm acquired with a time interval of 45 sec.
(AVI)

S2 Movie Intranuclear MT formation during M I. Time lapse imaging was performed with spermatocytes expressing GFP-βTub56D and His2Av-mRFP. MIPs of image stacks with 16 sections spaced by 500 nm and acquired at 10 second intervals were generated after deconvolution. The period after NEBD I when intranuclear MT formation starts is presented. Two prominent MT bundles formed at a distance from intranuclear spindle poles and chromosomes are marked.
(AVI)

S3 Movie Kinetochore tracking during M I. Time lapse imaging was performed with spermatocytes expressing Mis12-EGFP and His2Av-mRFP. MIPs of image stacks with 45 sections spaced by 300 nm and acquired at 10 second intervals were generated after deconvolution. The image sequence is repeated twice, the first time without annotations, the second time with chromosome identity indicated by KT color (chr X bright red, chr Y light red, chr 2 bright and light yellow, chr 3 bright and light blue, chr 4 bright and light green) and comet tails reporting V_KT_ with a color code from slow (blue) to fast (red). Scale bar = 3 μm.
(AVI)

S4 Movie Identification of chromosomes during M I. The m/a transition is shown after time lapse imaging with a spermatocyte expressing Mis12-EGFP and His2Av-mRFP. MIPs of image stacks with 40 sections spaced by 300 nm and acquired at 10 second intervals were generated after deconvolution. The image sequence is repeated multiple times. Repeats 1 and 2 lack annotations. In repeats 3 and 4, the two KTs of a bivalent are connected by a green line and KT color indicates chromosome identity (chr X bright red, chr Y light red, chr 2 bright and light yellow, chr 3 bright and light blue, chr 4 bright and light green). In repeats 5 and 6, additionally the euchromatic portion of the X chromosome is highlighted during anaphase. In repeats 7 and 8, isosurfaces of the Mis12-EGFP signals at KTs are shown with color indicating the intensity sum of the included pixel intensity. The KT of chr Y has maximal intensity, as revealed by its red color. Image rotations in the final part demonstrate that the KTs of the small chr 4 (displayed in bright and light green) and chr Y have minimal His2Av-mRFP signal associated. Identification of chr 2 and 3 is based on features apparent during prometaphase, which is not included in this movie.
(AVI)

S5 Movie Progression through M I after Spc105 depletion. Time lapse imaging was performed with spermatocytes expressing Cid-EGFP and His2Av-mRFP after spermatocyte-specific Spc105 depletion. MIPs of image stacks with 49 sections spaced by 300 nm and acquired at 10 second intervals were generated after deconvolution. Chromosome identity is indicated by KT color (chr X bright red, chr Y light red, chr 2 bright and light yellow, chr 3 bright and light blue, chr 4 bright and light green). Scale bar = 3 μm.
(AVI)

S6 Movie Progression through M I after colcemid addition. Time lapse imaging was performed with spermatocytes expressing Cid-EGFP and His2Av-mRFP after addition of the MT inhibitor colcemid. MIPs of images stacks with 31 sections spaced by 500 nm and acquired at 10 second intervals were generated after deconvolution. Scale bar = 5 μm.
(AVI)

S7 Movie KT tracking during M I in *mnm* mutants. Time lapse imaging was performed with *mnm* mutant spermatocytes expressing Cid-EGFP and His2Av-mRFP. MIPs of images stacks with 34 sections spaced by 300 nm and acquired at 10 second intervals were generated after deconvolution and time normalization. Chromosome identity is indicated by KT color (chr X bright red, chr Y light red, chr 2 bright and light yellow, chr 3 bright and light blue, chr 4 bright and light green) and comet tails report V_KT_ with a color code (scale setting as in S2 Movie). At the start of the movie in prometaphase A, the KTs of chrY and 4 are not already within the image stack. Moreover, the chr3 bivalent (blue KTs) is not yet disrupted into univalents like the chr 2 bivalent (yellow KTs). However the differentiation of the large autosomes remains tentative in this cell. The periods with evident stable separation of sister KTs (longer than three consecutive frames) are indicated by marking both sister KTs with a spot. Scale bar = 3 μm.
(AVI)

S8 Movie KT tracking during M I in *tef* mutants. Time lapse imaging was performed with *tef* mutant spermatocytes expressing Cid-EGFP and His2Av-mRFP. MIPs of images stacks with 25 sections spaced by 500 nm and acquired at 10 second intervals were generated. The image sequence is repeated three times. In repeat 1, only the KTs of the XY bivalent which behaves normally in *tef* mutant are marked by KT color (chr X bright red, chr Y light red) and comet tails. In the next two repeats, the two sister KTs within three distinct additional KTs are marked by color as well, starting at the onset of the metaphase-like period. Green sister KTs represent those of a chr 4 univalent where sister KTs are transiently separated during exit from M I after bi-orientation during prometaphase. Blue sister KTs indicated a comparable case revealed by a univalent of one of the large autosomes. Yellow sister KTs indicate those of the other chr4 univalent where bi-orientation and separation does not occur. In the last repeat the His2Av-mRFP channel is omitted. Scale bar = 3 μm.
(AVI)

S9 Movie Fzy/Cdc20 depletion results in a delay during M I. Spermatocyte-specific Fzy/Cdc20 depletion was performed in spermatocytes expressing Cid-EGFP and His2Av-mRFP. As revealed by time lapse imaging, this depletion resulted in an arrest in M I for about 5 hours followed by an exit from the arrest. MIPs generated from image stacks with 24 sections spaced by 800 nm acquired at 90 second intervals containing a somatic cyst cell and eleven spermatocytes of a cyst are displayed.
(AVI)
