# Supplementary material for: Dynamics and control of sister kinetochore behavior during the meiotic divisions in Drosophila spermatocytes
Source: PLoS Genet. 2018 May 7;14(5):e1007372. doi: 10.1371/journal.pgen.1007372 (PMC5957430; doi:10.1371/journal.pgen.1007372)
Supplement: S1 Table — (PDF) [file pgen.1007372.s001.pdf]

S1 Table. *Drosophila melanogaster* lines

Genotype	Reference
w; P{w^{+}, gHis2Av-mRFP} II.2* or *III.1*	(Schuh et al., 2007)
w; P{w^{+}, gCid-EGFP-Cid} II.1* or *III.1*	(Schuh et al., 2007)
w; P{w^{+}, gi2xtdTomato-Cenp-C} II.3 (CyO* floating*)*	(Althoff et al., 2012)
w; P{w^{+}, gSpc105-EGFP} II.1*	(Schittenhelm et al., 2009)
w; P{w^{+}, gMis12-EGFP} II.2*	(Schittenhelm et al., 2007)
w; P{w^{+}, gEGFP-Nuf2} III.1*	(Schittenhelm et al., 2007)
w; P{w^{+}, gEGFP-Bub3} II.1*	(Pandey et al., 2007)
w; P{w^{+}, UbiP-GFP[S65T]-ßTub56D} (17-1)*	(Inoue et al., 2004)<br>DGRC Kyoto # 109603
w; mnm[Z3-5578]/TM6B, Tb $Antp^{Hu}$*	(Thomas et al., 2005)
w; mnm[Z3-3298]/TM3, Ser*	(Thomas et al., 2005)
w; snm[Z3-0317]/TM3, Ser*	(Thomas et al., 2005)
w; snm[Z3-2138]/TM6B, Tb $Antp^{Hu}$*	(Thomas et al., 2005)
w; cn tef[Z2-4169] bw/SM1, Cy*	(Tomkiel et al., 2001)
w; cn tef[Z2-3455] bw/SM1, Cy*	(Tomkiel et al., 2001)
w; mad2[GE22825]*	(Buffin et al., 2007)
y w^{67c23}; mad2[EY21687] (TM3, Sb Ser floating*)*	Bloomington *Drosophila* Stock Center #22495
C(1; Y)1, y v f B: y+/C(1)RM, y^{2} su(w^{a}) w^{a}	Bloomington *Drosophila* Stock Center #700
w; P{lacO.256x, hsp26-SIP1, hsp70-mini-w+}55B*	(Danzer and Wallrath, 2004)
w; P{w^{+}, Hsp83-GFP.lacI}2, P{w^{+}, His2Av-mRFP} II.1*	Bloomington *Drosophila* Stock Center #25377
w; P{w^{+}, bamP-GAL4-VP16} III*	(Chen and McKearin, 2003)
w; P{w^{+}, matα4-GAL4-VP16}V2H*	(Hacker and Perrimon, 1998)
y sc v; UASt-Spc105-shmiR[TRiP.HMS01752]attP2/TM3, Sb*	(Ni et al., 2011)<br>Bloomington *Drosophila* Stock Center #38534
w; UASt-fzy-RNAi[KK101352]*	(Dietzl et al., 2007)<br>Vienna *Drosophila* Resource Center #105114

Althoff, F., Karess, R.E. and Lehner, C.F. (2012). Spindle checkpoint-independent inhibition of mitotic chromosome segregation by Drosophila Mps1. *Mol Biol Cell* **23**, 2275-2291.

Buffin, E., Emre, D. and Karess, R.E. (2007). Flies without a spindle checkpoint. *Nat Cell Biol* **9**, 565-572.

Chen, D. and McKearin, D. (2003). Dpp signaling silences bam transcription directly to establish asymmetric divisions of germline stem cells. *Curr Biol* **13**, 1786-1791.

Danzer, J.R. and Wallrath, L.L. (2004). Mechanisms of HP1-mediated gene silencing in Drosophila. *Development* **131**, 3571-3580.

Dietzl, G., Chen, D., Schnorrer, F., Su, K.C., Barinova, Y., Fellner, M., Gasser, B., Kinsey, K., Oppel, S., Scheiblauer, S., et al. (2007). A genome-wide transgenic RNAi library for conditional gene inactivation in Drosophila. *Nature* **448**, 151-156.

Hacker, U. and Perrimon, N. (1998). DRhoGEF2 encodes a member of the Dbl family of oncogenes and controls cell shape changes during gastrulation in Drosophila. *Genes Dev* **12**, 274-284.

Inoue, Y.H., Savoian, M.S., Suzuki, T., Mathe, E., Yamamoto, M.T. and Glover, D.M. (2004). Mutations in orbit/mast reveal that the central spindle is comprised of two microtubule populations, those that initiate cleavage and those that propagate furrow ingression. *J Cell Biol* **166**, 49-60.

Ni, J.Q., Zhou, R., Czech, B., Liu, L.P., Holderbaum, L., Yang-Zhou, D., Shim, H.S., Tao, R., Handler, D., Karpowicz, P., et al. (2011). A genome-scale shRNA resource for transgenic RNAi in Drosophila. *Nat Methods* **8**, 405-407.

Pandey, R., Heeger, S. and Lehner, C.F. (2007). Rapid effects of acute anoxia on spindle kinetochore interactions activate the mitotic spindle checkpoint. *J Cell Sci* **120**, 2807-2818.

Schittenhelm, R.B., Chaleckis, R. and Lehner, C.F. (2009). Essential functional domains and intrakinetochore localization of Drosophila Spc105. *EMBO J.* **28**, 2374-2386.

Schittenhelm, R.B., Heeger, S., Althoff, F., Walter, A., Heidmann, S., Mechtler, K. and Lehner, C.F. (2007). Spatial organization of a ubiquitous eukaryotic kinetochore protein network in Drosophila chromosomes. *Chromosoma* **116**, 385-402.

Schuh, M., Lehner, C.F. and Heidmann, S. (2007). Incorporation of Drosophila CID/CENP-A and CENP-C into centromeres during early embryonic anaphase. *Curr Biol* **17**, 237-243.

Thomas, S.E., Soltani-Bejnood, M., Roth, P., Dorn, R., Logsdon, J.M., Jr. and McKee, B.D. (2005). Identification of two proteins required for conjunction and regular segregation of achiasmate homologs in Drosophila male meiosis. *Cell* **123**, 555-568.

Tomkiel, J.E., Wakimoto, B.T. and Briscoe, A., Jr. (2001). The teflon gene is required for maintenance of autosomal homolog pairing at meiosis I in male Drosophila melanogaster. *Genetics* **157**, 273-281.
