# Supplementary material for: Dynamics and control of sister kinetochore behavior during the meiotic divisions in Drosophila spermatocytes
Source: PLoS Genet. 2018 May 7;14(5):e1007372. doi: 10.1371/journal.pgen.1007372 (PMC5957430; doi:10.1371/journal.pgen.1007372)
Supplement: S2 Table — (PDF) [file pgen.1007372.s002.pdf]

S2 Table. Sample genotypes

Fig 1A, B	w* / Y; P{w^+, gCid-EGFP-Cid} II.1, P{w^+, gHis2Av-mRFP} II.2 / CyO, P{ry^+, ftz-lacZ}; P{w^+, bamP-GAL4-VP16} III
Fig 1C	control:<br>w* / Y; P{w^+, gCid-EGFP-Cid} II.1, P{w^+, gHis2Av-mRFP} II.2, P{w^+, matα-GAL4-VP16}V2H / CyO<br><br>mad2:<br>w* / Y; P{w^+, gCid-EGFP-Cid} II.1, P{w^+, gHis2Av-mRFP} II.2, P{w^+, matα-GAL4-VP16}V2H / CyO or Sp; mad2[GE22825] / mad2[EY21687]
Fig 2A-D	w* / Y; P{w^+, UbiP-GFP[S65T]-ßTub56D} (17-1), P{w^+, gi2xtdTomato-Cenp-C} II.3 / CyO, P{Dfd-GMR-nvYFP}2
Fig 3A-D	w* / Y; P{w^+, gCid-EGFP-Cid} II.1, P{w^+, gHis2Av-mRFP} II.2 / CyO, P{ry^+, ftz-lacZ}; UASt-Spc105-shmiR[TRiP.HMS01752]attP2 / P{w^+, bamP-GAL4-VP16} III
Fig 3E	control:<br>w* / Y; P{w^+, gMis12-EGFP} II.2, P{w^+, gHis2Av-mRFP} II.2 / CyO, P{ry^+, ftz-lacZ}<br><br>Spc105 RNAi:<br>w* / Y; P{w^+, gMis12-EGFP} II.2, P{w^+, gHis2Av-mRFP} II.2 ; UASt-Spc105-shmiR[TRiP.HMS01752]attP2 / P{w^+, bamP-GAL4-VP16 III}
Fig 3F	control:<br>w* / Y; P{w^+, gEGFP-Bub3} II.1, P{w^+, gHis2Av-mRFP} II.2 ; MKRS/TM6B, Tb, $Antp^{Hu}$<br><br>Spc105 RNAi:<br>w* / Y; P{w^+, gEGFP-Bub3} II.1, P{w^+, gHis2Av-mRFP} II.2 ; UASt-Spc105-shmiR[TRiP.HMS01752]attP2 / P{w^+, bamP-GAL4-VP16} III<br><br>for scatter plot:<br>control:<br>w* / Y; P{w^+, gCid-EGFP-Cid} II.1, P{w^+, gHis2Av-mRFP} II.2 / CyO, P{ry^+, ftz-lacZ}; P{w^+, bamP-GAL4-VP16} III<br><br>Spc105 RNAi:<br>w* / Y; P{w^+, gCid-EGFP-Cid} II.1, P{w^+, gHis2Av-mRFP} II.2 / CyO, P{ry^+, ftz-lacZ}; UASt-Spc105-shmiR[TRiP.HMS01752]attP2 / P{w^+, bamP-GAL4-VP16} III
Fig 3G	control:<br>w* / Y; P{w^+, gCid-EGFP-Cid} II.1, P{w^+, gHis2Av-mRFP} II.2 / CyO, P{ry^+, ftz-lacZ}; P{w^+, bamP-GAL4-VP16} III<br><br>Spc105 RNAi:<br>w* / Y; P{w^+, gCid-EGFP-Cid} II.1, P{w^+, gHis2Av-mRFP} II.2 / CyO, P{ry^+, ftz-lacZ}; UASt-Spc105-shmiR[TRiP.HMS01752]attP2 / P{w^+, bamP-GAL4-VP16} III

Fig 3H	Spc105 RNAi:<br>w* / Y; P{w^{+}, gCid-EGFP-Cid} II.1, P{w^{+}, gHis2Av-mRFP} II.2 / CyO, P{ry^{+}, ftz-lacZ}; UASt-Spc105-shmiR[TRiP.HMS01752]attP2 / P{w^{+}, bamP-GAL4-VP16} III<br><br>colcemid:<br>w* / Y; P{w^{+}, gCid-EGFP-Cid} II.1, P{w^{+}, gHis2Av-mRFP} II.2 / CyO, P{ry^{+}, ftz-lacZ}; P{w^{+}, bamP-GAL4-VP16} III
Fig 4	w* / Y; P{w^{+}, gMis12-EGFP} II.2, P{w^{+}, gHis2Av-mRFP} II.2 / CyO, P{ry^{+}, ftz-lacZ}
Fig 5A, B	w* / Y; P{w^{+}, gCid-EGFP-Cid} II.1, P{w^{+}, gHis2Av-mRFP} II.2 / +; mnm[Z3-3298] / mnm[Z3-5578]
Fig 5C	control:<br>w* / Y; P{w^{+}, gCid-EGFP-Cid} II.1, P{w^{+}, gHis2Av-mRFP} II.2 / CyO, P{ry^{+}, ftz-lacZ}; P{w^{+}, bamP-GAL4-VP16} III<br><br>Spc105 RNAi:<br>w* / Y; P{w^{+}, gCid-EGFP-Cid} II.1, P{w^{+}, gHis2Av-mRFP} II.2 / CyO, P{ry^{+}, ftz-lacZ}; UASt-Spc105-shmiR[TRiP.HMS01752]attP2 / P{w^{+}, bamP-GAL4-VP16} III<br><br>mnm:<br>w* / Y; P{w^{+}, gCid-EGFP-Cid} II.1, P{w^{+}, gHis2Av-mRFP} II.2 / +; mnm[Z3-3298] / mnm[Z3-5578]<br><br>Spc105 RNAi mnm:<br>w* / Y; P{w^{+}, gCid-EGFP-Cid} II.1, P{w^{+}, gHis2Av-mRFP} II.2 / CyO, P{ry^{+}, ftz-lacZ}; P{w^{+}, bamP-GAL4-VP16} III, mnm[Z3-3298]/ UASt-Spc105-shmiR[TRiP.HMS01752]attP2, mnm[Z3-5578]<br><br>Spc105 RNAi snm:<br>w* / Y; P{w^{+}, gCid-EGFP-Cid} II.1, P{w^{+}, gHis2Av-mRFP} II.2 / CyO, P{ry^{+}, ftz-lacZ}; P{w^{+}, bamP-GAL4-VP16} III, snm[Z3-0317]/ UASt-Spc105-shmiR[TRiP.HMS01752]attP2, snm[Z3-2138]
Fig 6A	w* / Y; P{w^{+}, gCid-EGFP-Cid} II.1, P{w^{+}, gHis2Av-mRFP} II.2 / CyO, P{ry^{+}, ftz-lacZ}; P{w^{+}, bamP-GAL4-VP16} III
Fig 6B	control:<br>w* / Y; P{w^{+}, gCid-EGFP-Cid} II.1, P{w^{+}, gHis2Av-mRFP} II.2 / CyO, P{ry^{+}, ftz-lacZ}; P{w^{+}, bamP-GAL4-VP16} III<br><br>Spc105 RNAi:<br>w* / Y; P{w^{+}, gCid-EGFP-Cid} II.1, P{w^{+}, gHis2Av-mRFP} II.2 / CyO, P{ry^{+}, ftz-lacZ}; UASt-Spc105-shmiR[TRiP.HMS01752]attP2 / P{w^{+}, bamP-GAL4-VP16} III
Fig 6C-G	w* / Y; P{w^{+}, gMis12-EGFP} II.2, P{w^{+}, gHis2Av-mRFP} II.2 / CyO, P{ry^{+}, ftz-lacZ}
Fig 7A, B	w* / Y; P{w^{+}, gCid-EGFP-Cid} II.1, P{w^{+}, gHis2Av-mRFP} II.2 / +; mnm[Z3-3298] / mnm[Z3-5578]
Fig 7C, D	w* / Y; cn tef[Z2-4169] bw / cn tef[Z2-3455] bw; P{w^{+}, gCid-EGFP-Cid} III, P{w^{+}, gHis2Av-mRFP} III.1

Fig 7E	control:<br>w* / Y; P{w^{+}, gCid-EGFP-Cid} II.1, P{w^{+}, gHis2Av-mRFP} II.2 / CyO, P{ry^{+}, ftz-lacZ}; P{w^{+}, bamP-GAL4-VP16} III<br><br>mnm:<br>w* / Y; P{w^{+}, gCid-EGFP-Cid} II.1, P{w^{+}, gHis2Av-mRFP} II.2 / +; mnm[Z3-3298] / mnm[Z3-5578]<br><br>tef:<br>w* / Y; cn tef[Z2-4169] bw / cn tef[Z2-3455] bw; P{w^{+}, gCid-EGFP-Cid} III, P{w^{+}, gHis2Av-mRFP} III.1<br><br>fzy RNAi:<br>w* / Y; P{w^{+}, gCid-EGFP-Cid}II.1, P{w^{+}, gHis2Av-mRFP}II.2 / UASt-fzy-RNAi[KK101352]; P{w^{+}, bamP-GAL4-VP16} III/+<br><br>mnm fzy RNAi:<br>w* / Y; P{w^{+}, gCid-EGFP-Cid} II.1, P{w^{+}, gHis2Av-mRFP} II.2 / UASt-fzy-RNAi[KK101352]; P{w^{+}, bamP-GAL4-VP16} III, mnm[Z3-3298] / mnm[Z3-5578]
S2 Fig A	w* / Y; UbiP{GFP(S65T)-ßTub56D} 17-1, P{w^{+}, gHis2Av-mRFP} II.2/CyO
S2 Fig B, C	w* / Y; P{w^{+}, UbiP-GFP[S65T]-ßTub56D} (17-1), P{w^{+}, gi2xtdTomato-Cenp-C} II.3 / CyO, P{Dfd-GMR-nvYFP}2
S3 Fig	w* / Y; P{w^{+}, UbiP-GFP[S65T]-ßTub56D} (17-1), P{w^{+}, gi2xtdTomato-Cenp-C} II.3 / CyO, P{Dfd-GMR-nvYFP}2
S4 Fig A,D	w* / Y; P{w^{+}, gHis2Av-mRFP} II.2/ P{w^{+}, gSpc105-EGFP} II.1
S4 Fig B, E	w* / Y; P{w^{+}, gHis2Av-mRFP} II.2/ P{w^{+}, gMis12-EGFP} II.2
S4 Fig C, F	w* / Y; P{w^{+}, gHis2Av-mRFP} II.2/+; P{w^{+}, gEGFP-Nuf2} III.1/+
S5 Fig A-C, E	XY:<br>w* / Y; P{w^{+}, gCid-EGFP-Cid} II.1, P{w^{+}, gHis2Av-mRFP} II.2/CyO<br><br>X0:<br>w* / 0 ; P{w^{+}, gCid-EGFP-Cid} II.1, P{w^{+}, gHis2Av-mRFP} II.2 / +
S5 Fig D	w* / Y; P{w^{+}, Hsp83-GFP.lacI}2, P{w^{+}, His2Av-mRFP}II.1 / P{lacO.256x, hsp26-SIP1, hsp70-mini-w+}55B
S6 Fig A	w* / Y; P{w^{+}, gCid-EGFP-Cid} II.1, P{w^{+}, gHis2Av-mRFP} II.2 / CyO, P{ry^{+}, ftz-lacZ}; P{w^{+}, bamP-GAL4-VP16} III
S6 Fig B, C	w* / Y; P{w^{+}, gCid-EGFP-Cid} II.1, P{w^{+}, gHis2Av-mRFP} II.2 / CyO, P{ry^{+}, ftz-lacZ}; UASt-Spc105-shmiR[TRiP.HMS01752]attP2 / P{w^{+}, bamP-GAL4-VP16} III
S6 Fig D	Spc105 RNAi:<br>w* / Y; P{w^{+}, gCid-EGFP-Cid} II.1, P{w^{+}, gHis2Av-mRFP} II.2 / CyO, P{ry^{+}, ftz-lacZ}; UASt-Spc105-shmiR[TRiP.HMS01752]attP2 / P{w^{+}, bamP-GAL4-VP16} III<br><br>colcemid:<br>w* / Y; P{w^{+}, gCid-EGFP-Cid} II.1, P{w^{+}, gHis2Av-mRFP} II.2 / CyO, P{ry^{+}, ftz-lacZ}; P{w^{+}, bamP-GAL4-VP16} III
S7 Fig A	w* / Y; UbiP{GFP(S65T)-ßTub56D} 17-1, P{w^{+}, gHis2Av-mRFP} II.2/CyO

S7 Fig B	*w* / Y; P{w$^+$, gEGFP-Bub3} II.1, P{w$^+$, gHis2Av-mRFP} II.2 ; MKRS/TM6B, Tb, Antp^{Hu}*
S7 Fig C	control and control + colcemid:<br>*w* / Y; P{w$^+$, gCid-EGFP-Cid} II.1, P{w$^+$, gHis2Av-mRFP} II.2, P{w$^+$, matα-GAL4-VP16}V2H / CyO*<br><br>*mad2* + colcemid:<br>*w* / Y; P{w$^+$, gCid-EGFP-Cid} II.1, P{w$^+$, gHis2Av-mRFP} II.2, P{w$^+$, matα-GAL4-VP16}V2H / CyO* or *Sp; mad2[GE22825] / mad2[EY21687]*
S7 Fig D	*w* / Y; P{w$^+$, gCid-EGFP-Cid} II.1, P{w$^+$, gHis2Av-mRFP} II.2 / CyO, P{ry$^+$, ftz-lacZ}; P{w$^+$, bamP-GAL4-VP16} III*
S8 Fig	*w*; P{w$^+$, gCid-EGFP-Cid} II.1*
S9 Fig	*w* / Y; P{w$^+$, gCid-EGFP-Cid} II.1, P{w$^+$, gHis2Av-mRFP} II.2 / CyO, P{ry$^+$, ftz-lacZ}; P{w$^+$, bamP-GAL4-VP16} III*
