# Supplementary material for: Dynamics and control of sister kinetochore behavior during the meiotic divisions in Drosophila spermatocytes
Source: PLoS Genet. 2018 May 7;14(5):e1007372. doi: 10.1371/journal.pgen.1007372 (PMC5957430; doi:10.1371/journal.pgen.1007372)
Supplement: S1 Text — (PDF) [file pgen.1007372.s004.pdf]

S1 Text. Chromosome identification during time lapse analysis of M I

Chromosome identification was largely based on His2Av-mRFP signals (S5 Fig and S3 movie). Chromosome 4, a small dot chromosome, was associated with minimal His2Av-mRFP signals. Similarly, the Y chromosome contained only very low amounts of His2Av-mRFP, presumably reflecting its unusual heterochromatic character. Moreover, the large block of centromere-proximal heterochromatin of the X chromosome, which is the only acrocentric among the *D. melanogaster* chromosomes, had also very low His2Av-mRFP levels in contrast to its distal euchromatic part. The bivalents formed by the large autosomes, chromosome 2 and 3, were represented by prominent His2Av-mRFP containing regions. In case of these large autosomes, each of the two KTs of a bivalent was associated with a comparable His2Av-mRFP domain, in contrast to the asymmetric XY bivalent.

Distinction of the two large metacentric autosomes, chromosomes 2 and 3, was most difficult but often possible based on characteristic differences in the pattern of His2Av-mRFP signal intensity within the pericentromeric regions (S5 Fig). One of the large autosome bivalents was characterized by His2Av-mRFP blobs close to the KTs that were especially prominent. These blobs were most evident during prometaphase. To distinguish whether the large autosome characterized by these prominent KT-proximal His2Av-mRFP blobs was chromosome 2 or 3, we analyzed spermatocytes that had a lacO repeat array on one of the two chromosome 2 homologs (S5 Fig). Moreover, these spermatocytes also expressed GFP-lacI-nls and His2Av-mRFP. Time lapse imaging revealed that the GFP-lacI-nls dot signal was present on the large autosome that did not display the prominent KT-proximal His2Av-mRFP blobs. Therefore, the large autosome characterized by these blobs appears to be chromosome 3.

Further help for chromosome identification came from the almost twofold higher intensity of the Cid-EGFP and Mis12-EGFP signals in the centromere of the Y chromosome [1]. While recently questioned [2], we performed additional signal intensity quantification with our time lapse data which clearly confirmed that Cid-EGFP signals were consistently increased specifically in the centromere of the Y chromosome (S5 Fig, S3 Movie). Quantitative analyses of XY spermatocytes indicated that the brightest Cid-EGFP dot was associated consistently with a

unique bivalent characterized by a prominent but asymmetric His2Av-mRFP domain, i.e. the XY bivalent (S5 Fig, S3 Movie). Moreover, analogous quantitative analyses of X0 spermatocytes revealed that they no longer had a Cid-EGFP dot that was significantly more intense than the other dots (S5 Fig). X0 spermatocytes were observed to progress through M I frequently with severe abnormalities, precluding a reliable assignment of centromeres to chromosomes. X0 spermatocytes cannot generate pi-RNAs from the Y chromosomal *Su(Ste)* repeat locus and therefore express the X-chromosomal *Ste* repeats, resulting in protein aggregation, defects during chromosome segregation and poor fertility. However, progression through M I was sufficiently normal in some X0 spermatocytes, allowing unequivocal identification of chromosomes and quantification of associated centromeric Cid-EGFP signals.

The recent discrepant conclusions concerning centromeric Cid levels on the sex chromosomes might reflect experimental limitations of the applied immuno-FISH approach [2]. Quantification of immunofluorescent signals can suffer from accessibility and fixation problems in particular when combined with DNA FISH. Moreover, the AATAC probe hybridizes to a chromosomal location that is quite distant from the Y centromere, which in turn is often closely associated in spermatocytes with the centromeres of the X and the fourth chromosome before NEBD I. As a result, assigning the AATAC FISH signal correctly to the Cid-EGFP dot of the Y centromere is difficult. However, tracking centromeres and chromosomes in live spermatocytes during M I permits a very reliable Y chromosome identification. Of note, as a consequence of the distinct separation between centromeres and rDNA loci on the X and Y chromosomes, the Y centromere is usually closer to the His2Av-mRFP positive X-chromosomal euchromatin than the X centromere. Moreover, Cid-EGFP dot intensities depend on their position along the z-axis. At times where the Y centromere is far more distant from the coverslip than other centromere signals, its higher Cid-EGFP level is not necessarily apparent.

1. Raychaudhuri N, Dubruille R, Orsi GA, Bagheri HC, Loppin B, Lehner CF. Transgenerational propagation and quantitative maintenance of paternal centromeres depends on Cid/Cenp-A presence in Drosophila sperm. PLoS Biol. 2012;10(12):e1001434.
2. Kwenda L, Collins CM, Dattoli AA, Dunleavy EM. Nucleolar activity and CENP-C regulate CENP-A and CAL1 availability for centromere assembly in meiosis. Development. 2016;143(8):1400-12.
