# Supplementary figures and images for: Dynamics and control of sister kinetochore behavior during the meiotic divisions in Drosophila spermatocytes
Source: PLoS Genet. 2018 May 7;14(5):e1007372. doi: 10.1371/journal.pgen.1007372 (PMC5957430; doi:10.1371/journal.pgen.1007372)

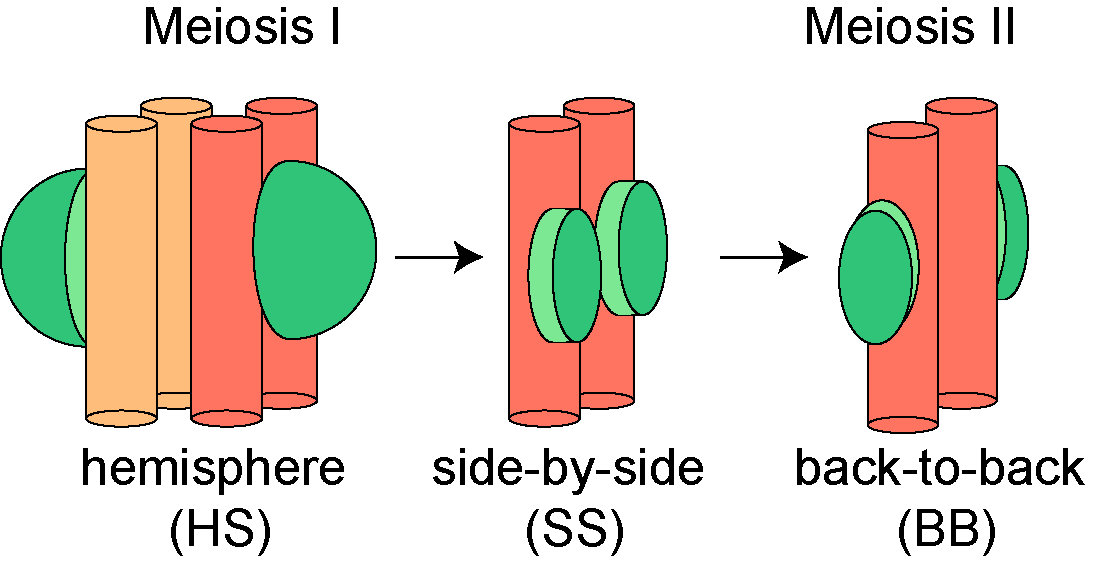

Supplement: S1 Fig — Based on serial sectioning and electron microscopy with Drosophila spermatocytes [18], a single hemispherical structure (HS) is present at the start of M I, composed of the two sister KTs, which cannot be distinguished. Later in M I, a side-by-side arrangement (SS) is observed with two closely associated KT discs (i.e., the two sister KTs in all likelihood). It is assumed that sister KTs eventually adopt a back-to-back organization (BB) with inner centromere chromatin in between. The precise timing of the HS to SS and SS to BB transitions and their molecular basis are unknown. (TIF) [file pgen.1007372.s005.tif]

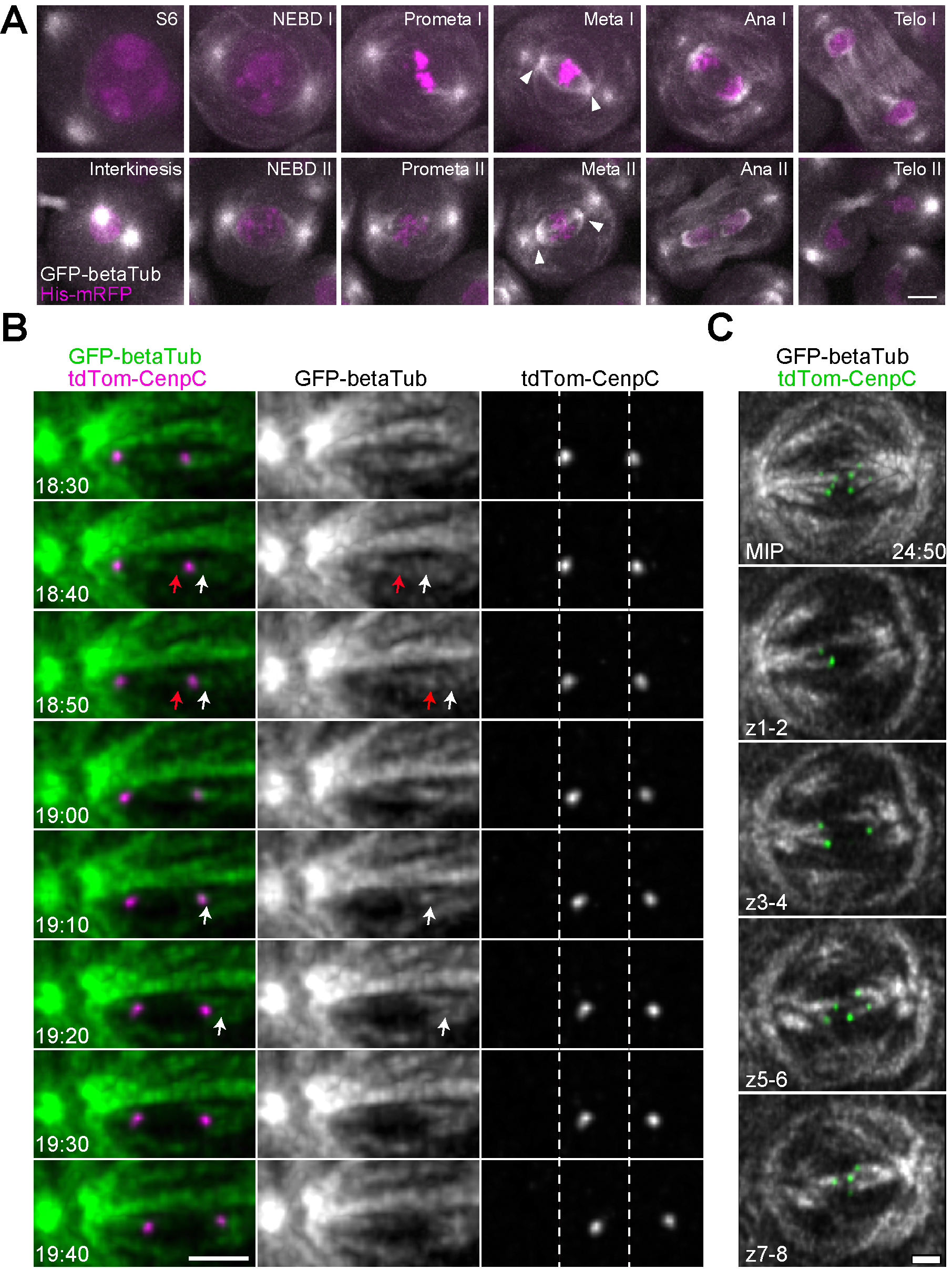

Supplement: S2 Fig — (A) The dynamics of spindle organization during the meiotic divisions was analyzed by time lapse imaging with spermatocytes expressing GFP-βTub56D (GFP-betaTub) and His2Av-mRFP (His-mRFP). Still frames showing a representative spermatocyte at the indicated stages. The intranuclear spindle poles which were most prominent during metaphase are indicated by arrowheads. (B) Congression of bivalents into the metaphase plate was analyzed by time lapse imaging with spermatocyte cysts expressing GFP-βTub56D and tdTomato-Cenp-C. High magnification views document the onset of the final congression of a bivalent into the metaphase I plate. Maximum intensity projections of only those optical sections containing the two KTs of the chosen bivalent are shown at the indicated time points (minutes:seconds after NEBD I). Dotted white lines mark the initial KT positions for reference. One centrosome and the associated intranuclear spindle pole are visible on the left side. Spindle axis is horizontal. Congression onset is accompanied by the apparent disappearance of the connections (red arrows) between the left spindle pole and the merotelically attached KT on the right, while its connections to the right spindle pole remain (white arrows). (C) Stably bi-oriented bivalents in metaphase I have KTs with end-on attachments to prominent MT bundles. A maximum intensity projection (MIP) of eight optical sections with 300 nm spacing is shown in the top panel, while MIPs of only two sections (section numbers as indicated) are displayed below. Scale bar = 3 μm. (TIF) [file pgen.1007372.s006.tif]

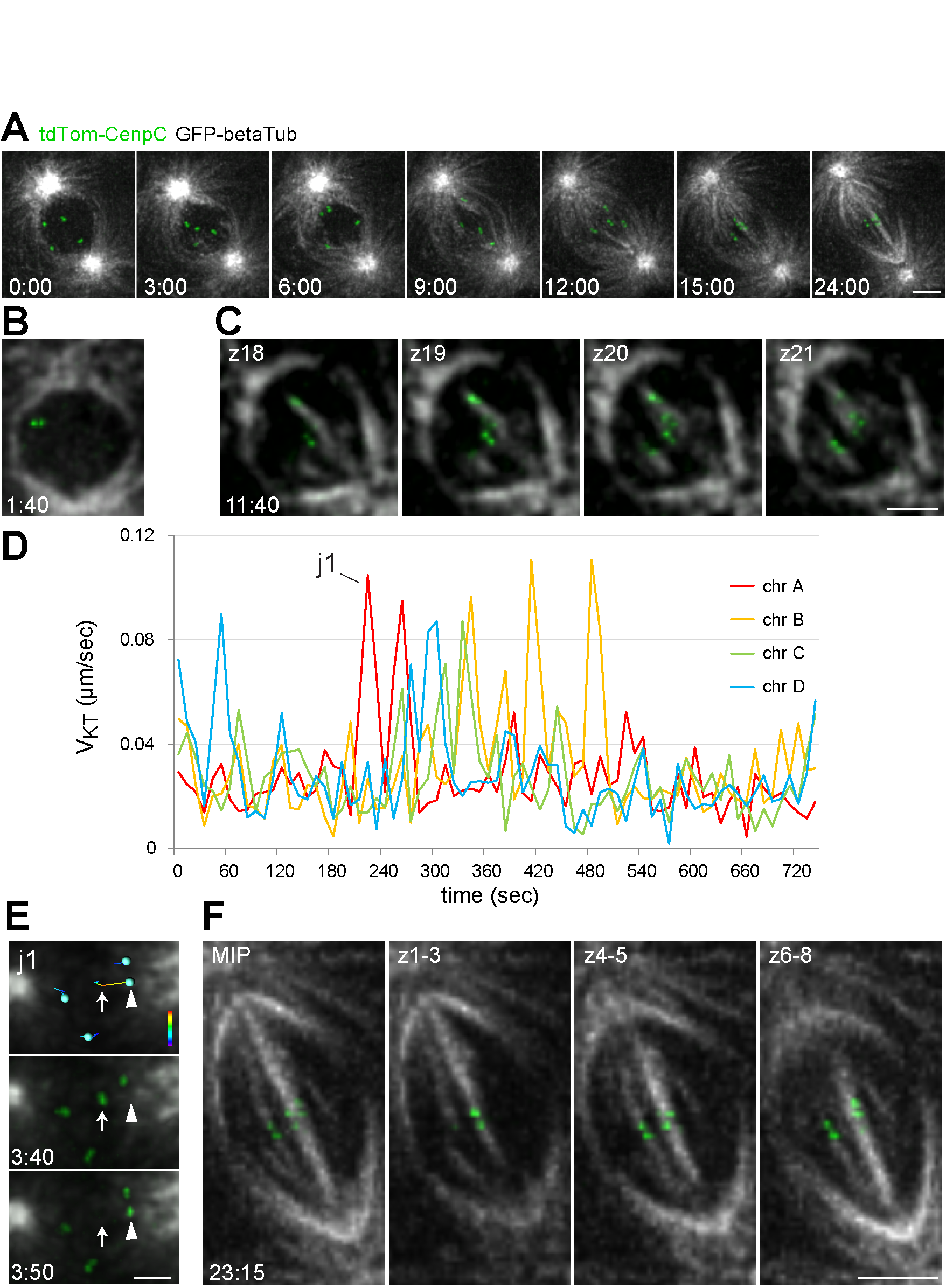

Supplement: S3 Fig — Time lapse imaging was performed with spermatocytes expressing tdTomato-Cenp-C and GFP-βTub56D. (A) Still frames at selected time points (minutes:seconds after NEBD II) illustrate progression until metaphase II in a representative spermatocyte. Each panel is a MIP of the z slices containing the spindle poles (3–4 optical sections with 300 nm spacing). Scale bar = 2 μm. (B) A single optical section documenting an example of a transient sister KT separation event during early prometaphase with an inter sister KT axis that is not oriented towards one of the spindle poles and without associated detectable MTs. (C) Later movements of KT pairs during prometaphase II can occur in association with MTs that are not necessarily oriented towards a spindle pole. Four consecutive z-sections are shown. Scale bar = 3 μm. (D) The four sister KT pairs were tracked in a representative cell from NEBD until metaphase, and the VKT values were plotted over time. The first KT jump event (j1) is documented in panel E. (E) The top panel includes an overlay with KT tracks over the preceding four time points (40 seconds) which include the KT jump indicated in panel D (j1). Track colors reflect VKT from slow (blue) to fast (red). The middle and bottom panels document localization of MTs and sister KT pairs before and after the jump, respectively. Arrows and arrowheads indicate the position of the KT that is leading during the jump, before and after the jump, respectively. Scale bar = 2 μm. (F) Stably bi-oriented dyads in metaphase have KT with end-on attachments to prominent MT bundles. Left most panel is a MIP comprising of eight optical sections with 300 nm spacing. MIPs of only a few sections (as indicated) are shown in the additional panels. Scale bar = 3 μm. (TIF) [file pgen.1007372.s007.tif]

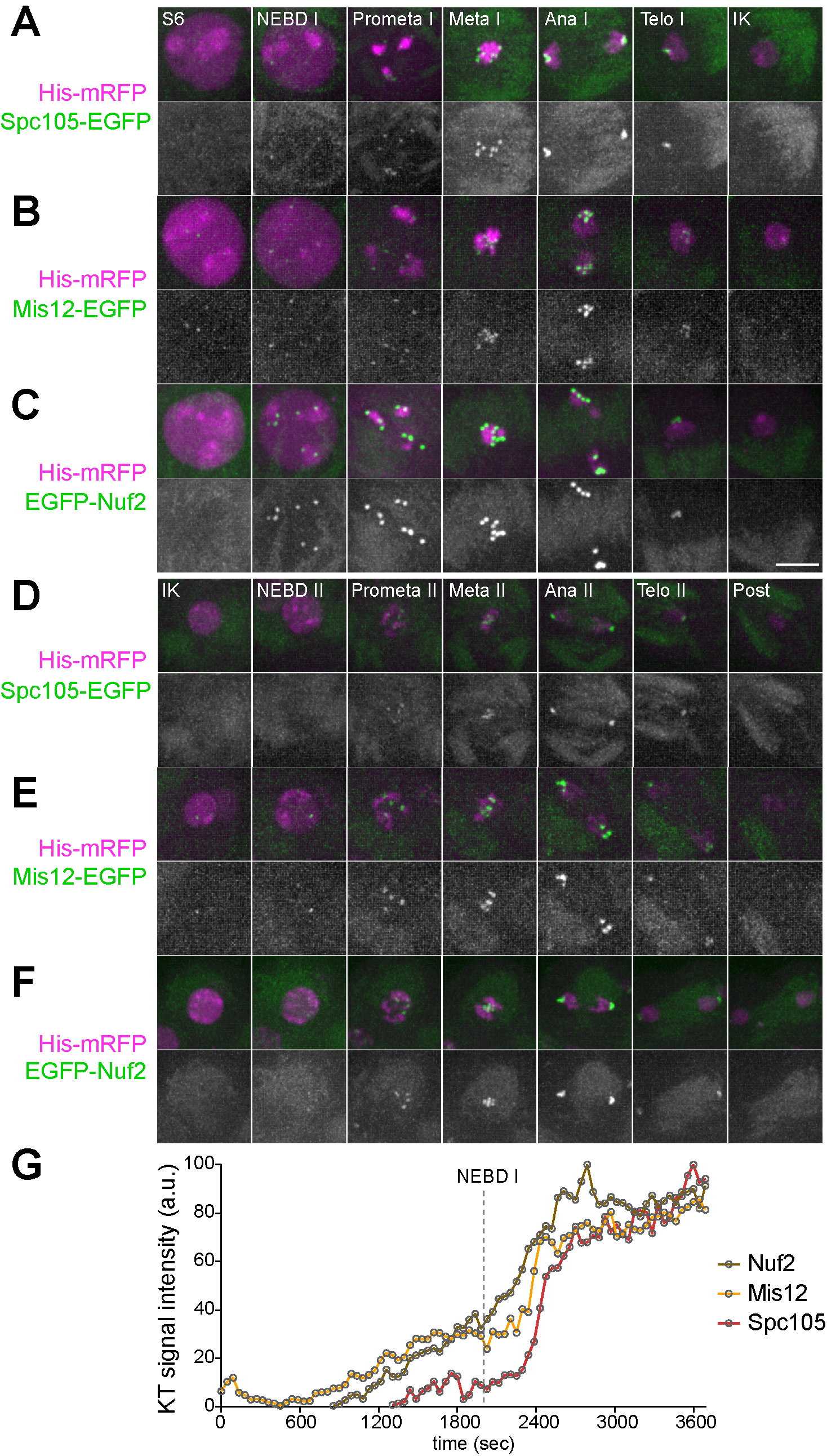

Supplement: S4 Fig — Time lapse imaging with spermatocytes was performed for the analysis of the meiotic localization of KMN network proteins (Nuf2, Spc105 and Mis12) fused to EGFP. Spermatocytes also expressed His2Av-mRFP for monitoring progression through the meiotic divisions. Representative spermatocytes are shown at the indicated stages: spermatocytes at stage S6 (S6), NEBD, prometaphase, metaphase, anaphase, telophase, interkinesis (IK), and postmeiotic interphase (Post). (A, D) Spc105-EGFP. (B, E) Mis12-EGFP. (C, F) Nuf2-EGFP. Scale bar = 5 μm. (G) EGFP signal intensities at KTs from representative cells during entry into M I. Maximal intensity was set to 100 arbitrary units (a.u.). NEBD was used for curve alignment. (TIF) [file pgen.1007372.s008.tif]

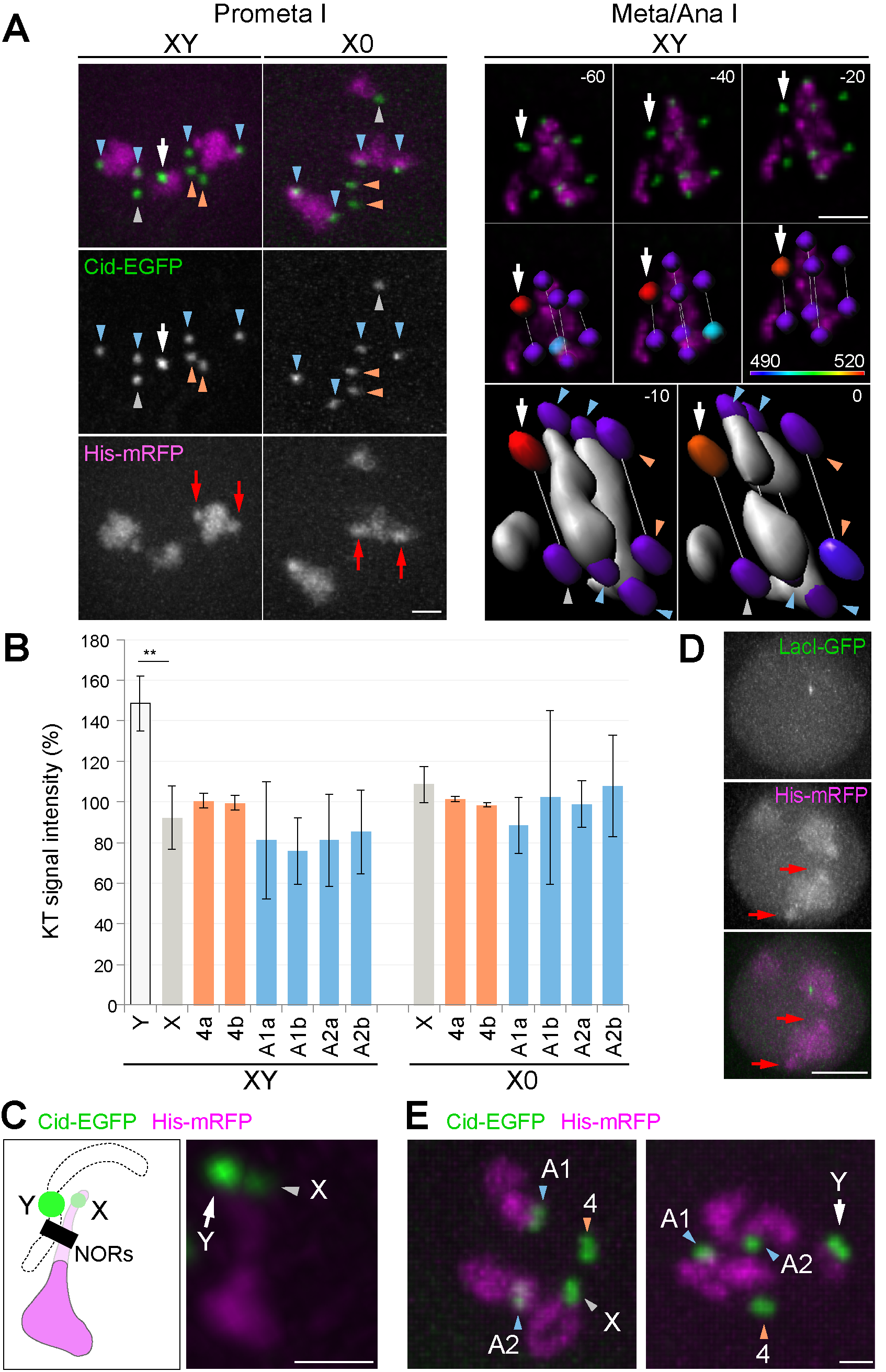

Supplement: S5 Fig — (A) Appearance of chromosomes and associated Cid-EGFP signals during M I in XY and X0 spermatocytes. After Cid-EGFP and His2Av-mRFP time lapse imaging, four distinct bivalents could be differentiated and identified in spermatocytes with a normal XY karyotype during prometaphase I (for further explanations see S1 Text). The two Cid-EGFP dots of the chromosome 4 bivalent are indicated by orange arrowheads, those of the large autosomes (chromosome 2 and 3) by blue arrowheads. The prominent His2Av-mRFP blobs next to the KTs of chromosome 3 are indicated by red arrows. The Cid-EGFP dot of the X chromosome is indicated with a grey arrowhead, that of the Y chromosome (absent in X0) with a white arrow. During the metaphase to anaphase transition in XY spermatocytes (Meta/Ana I) the Cid-EGFP dot on the Y chromosome has minimal associated His2Av-mRFP signals. Time (seconds) is indicated in the upper right corner with zero corresponding to the first anaphase frame. Cid-EGFP intensities quantified after spot segmentation are color-coded from low (blue) to high (red) in the middle and bottom rows. Amounts of associated His2Av-mRFP are visualized by the white isosurfaces in the bottom row. Scale bars = 2 μm. (B) Average intensity (+/- s.d.) of Cid-EGFP dots in XY (n = 4) and X0 spermatocytes (n = 3). While X, Y and 4th chromosomes were identified, the two large autosomes (A1 and A2) were not distinguished as either chromosome 2 or 3. In each cell, the average intensity of the two Cid-EGFP dots associated the chromosome 4 bivalent was set to 100%. The difference between the X and the other KTs was highly significant (p < 0.0017, t test). (C) Characteristic features of the XY bivalent. The Y chromosome contains only very low levels of His2Av-mRFP, and in the X chromosome His2Av-mRFP is primarily present in the centromere-distal euchromatic arm region. XY pairing is mediated by repeats within the rDNA loci of these chromosomes (NORs). Scale bar = 1 μm. (D) Time lapse imaging was per [file pgen.1007372.s009.tif]

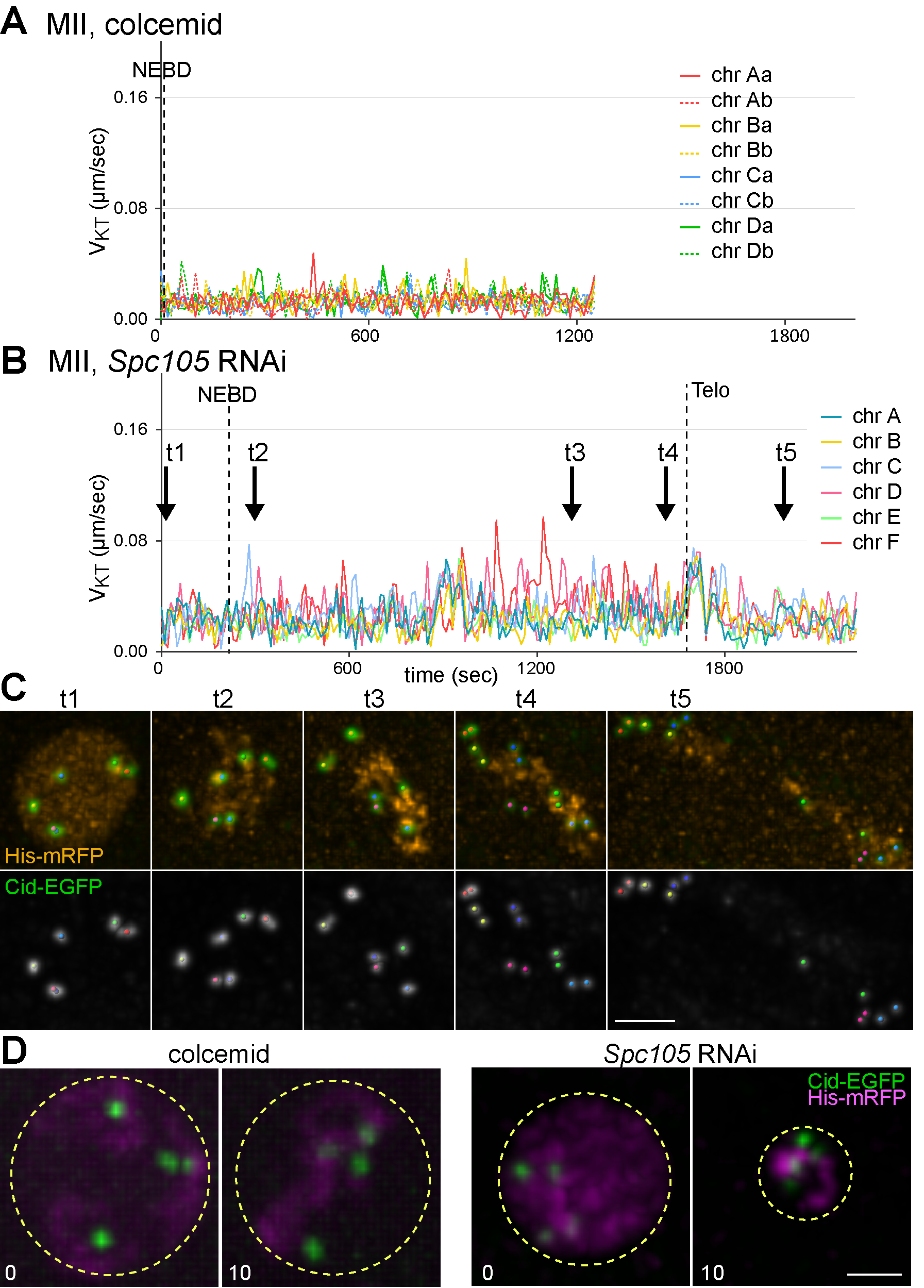

Supplement: S6 Fig — (A) Time lapse imaging was performed with spermatocytes expressing Cid-EGFP and His2Av-mRFP after addition of colcemid. All eight sister KTs in a representative spermatocyte were tracked during the first 20 minutes of M II. VKT values were plotted over time. (B,C) Time lapse imaging was performed with spermatocytes expressing Cid-EGFP and His2Av-mRFP after spermatocyte-specific Spc105 depletion. (B) One sister KT in each dyad was tracked during progression through M II and VKT values over time were plotted. Arrows (t1-t5) indicate the time points documented in (C). (C) Still frames at selected time points (see B) illustrate delayed sister KT separation and chromosome movements accompanying chromosome decondensation during exit from M II. Sister KTs co-segregated during these chromosome movements rather than move apart as during normal M II. (D) Still frames from the indicated times (minutes after NEBD) after treatment with colcemid or Spc105 depletion indicate that spindle assembly can drive congression of dyads into the central region in a KT-independent manner. Yellow circles indicate the regions occupied by chromosomes. Scale bars = 3 μm (C) and 2 μm (D). (TIF) [file pgen.1007372.s010.tif]

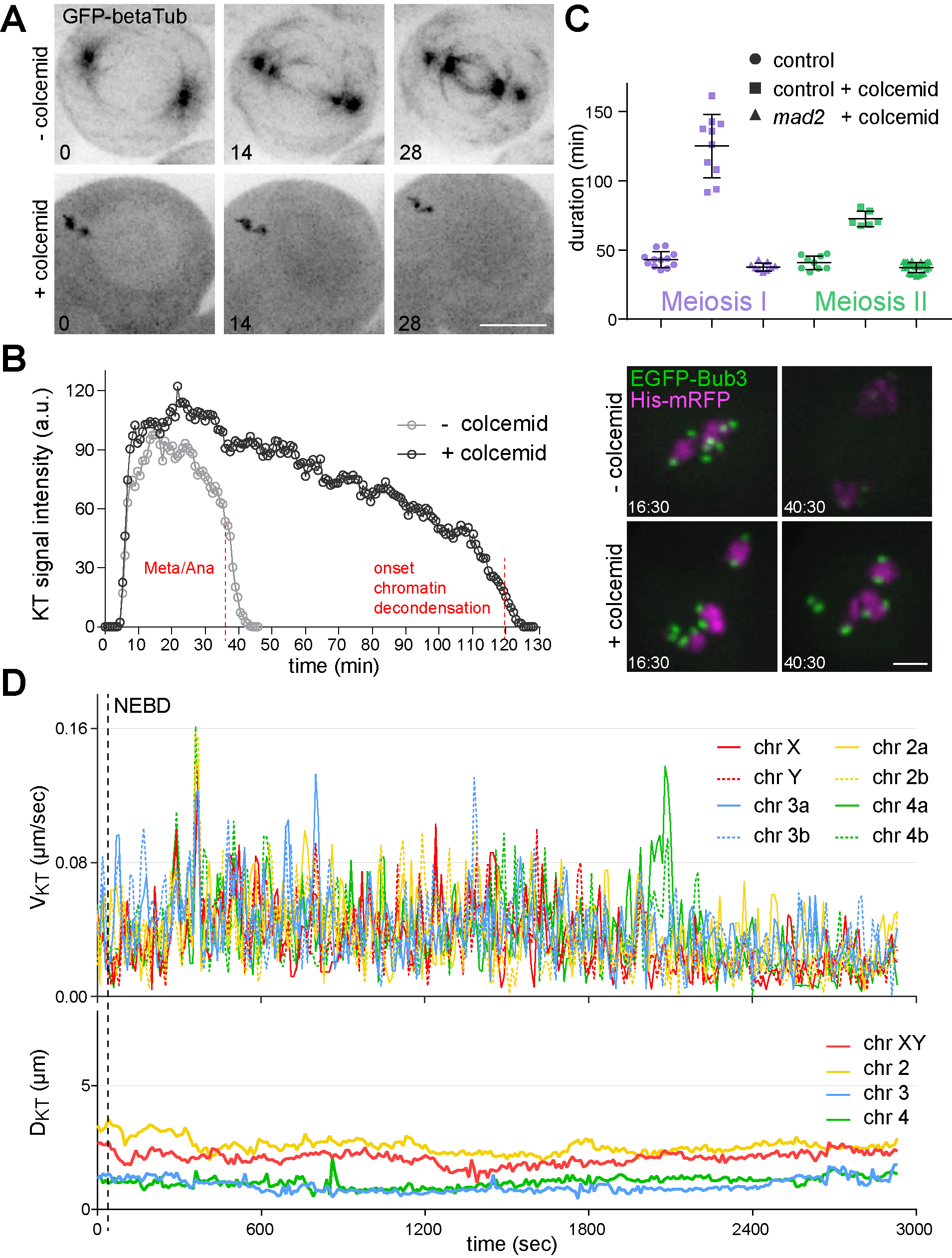

Supplement: S7 Fig — (A) Time lapse imaging with spermatocytes expressing GFP-βTub56D and His2Av-mRFP demonstrated that addition of colcemid prevents spindle formation effectively. In contrast, spindle assembly proceeds normally in mock-treated spermatocytes. Time (minutes) is indicated in the lower left corner with zero corresponding to NEBD I. Scale bar = 10 μm. (B) Colcemid delays the release of Bub3 from KTs and exit from M I. Spermatocytes expressing EGFP-Bub3 and His2Av-mRFP were used for time lapse imaging of progression through M I. In the absence of colcemid, EGFP-Bub3 signal intensities reach a peak at KTs in prometaphase and drop at a maximal rate in anaphase. In the presence of colcemid, EGFP-Bub3 disappearance from KTs and exit from M I are strongly delayed. Representative time points (minutes:seconds after NEBD) are illustrated by still frames. Analogous observations were made in all other spermatocytes analyzed (at least eight from two different cysts for each condition). Scale bar = 3 μm. (C) Colcemid induces a strong delay during M I and M II. Colcemid was added just before either NEBD I or NEBD II. The duration of the meiotic divisions (NEBD until cytokinesis onset) was determined in control and mad2 null mutant cells. Average durations +/- s.d. are from n ≥ 6 cells from at least six different testes. (D) Colcemid eliminates rapid KT jumps during prometaphase and the stretching apart of KTs that accompanies chromosome bi-orientation during unperturbed meiosis. Time lapse imaging was performed with spermatocytes expressing Cid-EGFP and His2Av-mRFP after addition of colcemid. All eight KTs in a representative spermatocyte were tracked during M I. VKT and DKT values were plotted over time. (TIF) [file pgen.1007372.s011.tif]

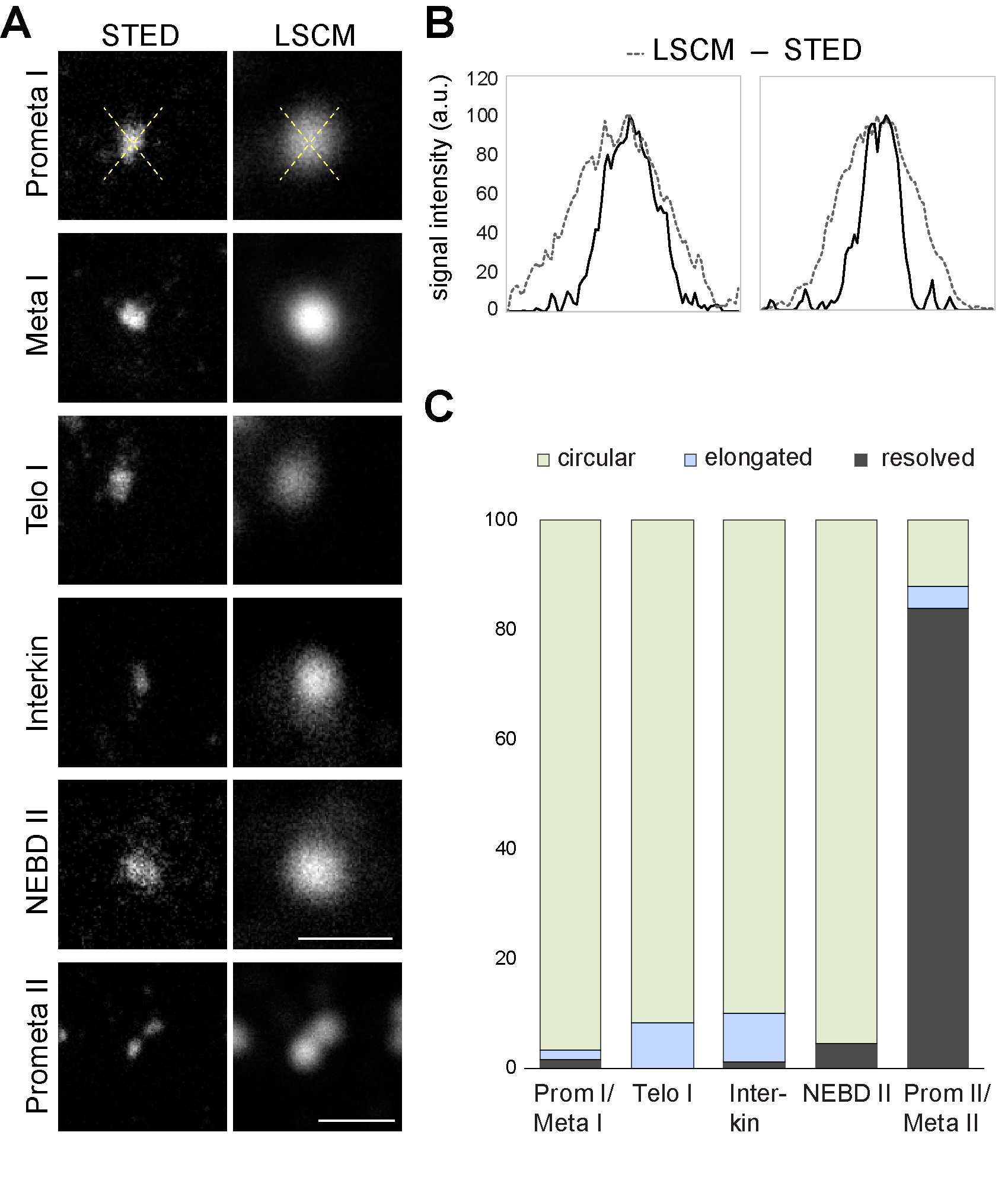

Supplement: S8 Fig — (A-C) Testis squash preparations of Cid-EGFP expressing spermatocytes were fixed and labeled with anti-GFP. Double labeling with a DNA stain (not displayed) allowed identification and staging of cells during the meiotic divisions. (A) Representative single optical sections through a given Cid-EGFP dot acquired by STED and confocal laser scanning (LSC) microscopy, respectively. All panels displayed at the same magnification except for prometa II. Scale bars = 1 μm. (B) Intensity curves along the yellow dotted lines in (A) confirm increased resolution of STED images. (C) Quantification of the fraction of Cid-EGFP dots with a circular (see for example (A) meta I), elongated (see for example (A) interkinesis) and resolved sisters (see for example (A) prometa II) appearance at the indicated stages. (TIF) [file pgen.1007372.s012.tif]

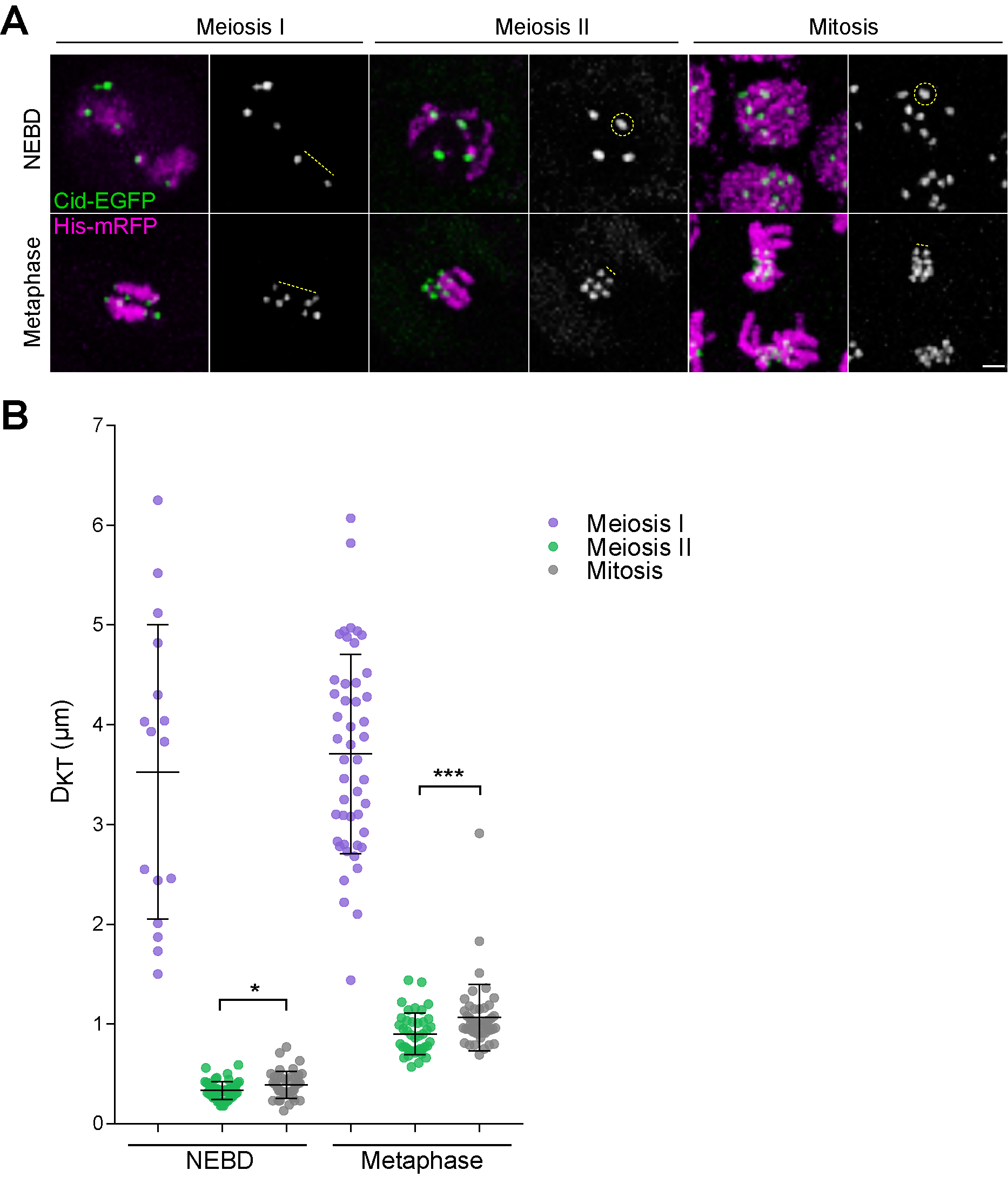

Supplement: S9 Fig — Identical settings were used for time lapse imaging with spermatocytes and embryos expressing Cid-EGFP and His2Av-mRFP. The distance between sister KTs was measured at NEBD and in metaphase. (A) Representative still frames are displayed. The two homologous centromere pairs are widely separated in bivalents during M I, as indicated by the yellow dotted line. In contrast, sister KTs cannot be resolved initially at the start of M II and mitosis, as indicated by the yellow dotted circles. Scale bar = 2 μm. (B) Dot plot representing individual measurements as well as average (+/- s.d.); at NEBD, n = 16 KT pairs (M I), 51 sister KT pairs (M II) and 42 sister KT pairs (mitosis); at metaphase, n = 48 KT pairs (M I), 42 sister KT pairs (M II) and 51 sister KT pairs (mitosis). Differences between average separation were statistically significant (t test): (*) p = 0.024, (***) p = 0.0058. (TIF) [file pgen.1007372.s013.tif]
